# Myeloid Cell Targeting Strategies Show Limited Durable Activity in the Breast Cancer Tumor Microenvironment and Do Not Enhance the Activity of Thermally Ablative Focused Ultrasound

**DOI:** 10.3390/cells15111035

**Published:** 2026-06-04

**Authors:** Carly M. Van Wagoner, Lydia E. Kitelinger, Matthew R. DeWitt, Claire A. Conarroe, AeRyon Kim, Aaron B. Streit, Richard J. Price, Timothy N. J. Bullock

**Affiliations:** 1 Department of Microbiology, Immunology, and Cancer Biology, University of Virginia, Charlottesville, VA 22908, USA; cmv5cx@virginia.edu; 2 Department of Pathology, University of Virginia, Charlottesville, VA 22908, USA; lep2gw@virginia.edu (L.E.K.); kyj2gv@virginia.edu (C.A.C.); svn7fz@virginia.edu (A.K.); saf7nj@virginia.edu (A.B.S.); 3 Department of Radiology & Medical Imaging, University of Virginia, Charlottesville, VA 22908, USA; nac7za@virginia.edu (M.R.D.); rjp2z@virginia.edu (R.J.P.)

**Keywords:** triple-negative breast cancer, myeloid depletion, TLR agonism, myeloid reprogramming, tumor microenvironment, tumor immunology, focused ultrasound

## Abstract

Triple-negative breast cancer (TNBC) is the most aggressive subtype of breast cancer (BrCa), owing to its lack of targetable receptors and resistance to chemical and molecularly targeted therapeutic approaches. While chemotherapy and surgical resection remain the standard of care, these interventions have significant side effects and varying patient outcomes. Thermally ablative focused ultrasound (T-FUS)—a non-invasive and non-ionizing therapy that utilizes targeted acoustic energy to debulk tumors—has displayed immunomodulatory effects in BrCa. However, T-FUS as a monotherapy has had limited clinical efficacy in TNBC due to the presence of anti-inflammatory immunosuppressive myeloid cells (isMCs). We hypothesized that the elimination of isMCs or initiating tumoricidal activity from them would lead to augmented activity of T-FUS. Thus, we interrogated the ability of myeloablative chemotherapies and antibodies; myeloid recruiting chemokine receptor blockade; and TLR agonists to remodel the tumor myeloid populations. Consistent with our previous studies, we found that while myeloablative chemotherapies decreased circulating isMCs, they had little impact on intratumoral isMCs. In contrast, antibodies targeting Ly6C and Ly6G ablated intratumoral isMCs and systemic isMCs, yet their effect was transient and was accompanied by a surprising depletion of T cells. While targeting CCR2, the dominant chemokine receptor for intratumoral isMC diminished a large subset of immunosuppressive cells within the TME; it also depleted T cells and dendritic cells. Contrary to previous studies, TLR stimulation failed to repolarize myeloid cells into a pro-inflammatory, tumoricidal phenotype but did lead to their depletion from the tumor microenvironment (TME) and mobilization of conventional dendritic cells to the draining lymph nodes. We therefore hypothesized that combining isMC depletion and TLR-driven immune activation would enhance FUS efficacy; however, this combinatorial regimen did not enhance overall survival or control tumor volume after T-FUS treatment. Thus, the BrCa TME is highly resistant to approaches intended to remodel the myeloid cell component which fail to synergize with T-FUS-mediated tumor ablation.

## 1. Introduction

Breast cancer (BrCa) is the most commonly diagnosed cancer among US women, with an estimated 316,950 new cases diagnosed in 2025 [1]. It is a highly heterogeneous disease commonly classified into clinical subtypes based on the expression of hormone receptors: estrogen (ER), progesterone (PR), and human epidermal growth factor receptor 2 (HER2) [2]. Triple-negative breast cancer (TNBC), defined by the absence of these receptors, accounts for approximately 15–20% of all BrCa cases and remains the only major subtype lacking targeted therapies [3]. TNBC is biologically aggressive and prone to early metastatic spread, which—combined with limited targeted treatment options—results in poor clinical outcomes and a five-year mortality rate approaching 40% [4,5,6,7,8].

Cytotoxic chemotherapy remains the standard of care for TNBC patients despite extensive efforts to discover actionable molecular targets. Current therapeutic advances include immune checkpoint inhibitors (ICIs) targeting the PD1/PD-L1 axis; however, PD-L1 expression is dynamic and spatially heterogeneous within tumors [9,10,11], and only 20–34% of TNBC cases meet PD-L1 expression criteria for treatment eligibility [12,13,14]. Poly(ADP-ribose) polymerase (PARP) inhibitors have shown promise, particularly in combination with ICIs [15,16,17] and chemotherapy [18,19,20], but their efficacy is often compromised by the emergence of resistance mechanisms [21]. Thus, despite meaningful progress in immunotherapy and targeted approaches, chemotherapy remains the standard of care as it provides the most broadly applicable therapeutic benefit across a disease landscape. These limitations have prompted growing interest in multimodal strategies to overcome TNBC’s profound immunologic barriers.

Building on this need, image-guided focused ultrasound (FUS) is a non-invasive and non-ionizing therapy capable of delivering targeted acoustic energy to debulk tumor through thermal necrosis (T-FUS) or mechanical destruction (histotripsy) [22,23,24,25,26,27,28,29,30,31,32,33,34,35,36]. In solid tumors, FUS can overcome immunological barriers posed by dense stroma, low antigen availability, and poor immune cell infiltration by inducing localized tumor injury and subsequent antigen release. The associated liberation of tumor antigens and immunostimulatory molecules—such as damage associated molecular patterns (DAMPs) and heat shock proteins (HSPs)—positions FUS as a potentially immunogenic intervention in settings where other therapies fall short.

A considerable barrier to immunity in TNBC is the presence of immunosuppressive myeloid cells (isMCs). These cells play a pivotal role in facilitating tumor growth, recurrence, and metastasis by inhibiting T cell proliferation and cytotoxic activity, supporting angiogenesis, and triggering metastatic dissemination [37]. In BrCa, elevated circulating and intratumoral isMCs have been linked to therapeutic resistance and are associated with metastatic disease and reduced survival [38,39]. Further, TNBC tumors have a greater level of isMC infiltration than non-TNBC subtypes, adding to the immunosuppressive and metastatic nature of this disease [40].

We previously demonstrated that T-FUS combined with gemcitabine (GEM) can drive T cell-dependent tumor control [41]. We hypothesized that the efficacy of this combination was in part due to the myeloablative effects of GEM. However, we subsequently found that GEM’s myeloablative effects are often incomplete or transient within the TME [42] suggesting that persistent isMCs may be a contributing barrier to achieving durable, abscopal responses following thermal ablation.

In this study, we used two preclinical models of TNBC to analyze three distinct pharmacologic and immunological strategies that have been demonstrated to modulate the myeloid compartment so as to overcome myeloid-mediated resistance: (1) systemic chemotherapy (GEM and Doxorubicin, DOX), (2) targeted antibody-mediated depletion/blockade (anti-Gr1, anti-Ly6G, and anti-CCR2 tumors) and (3) functional reprogramming via TLR agonism (CpG-B and poly-ICLC). Our goal was to determine whether relieving myeloid suppression—either by physical removal or phenotypic shifting—would synergize with T-FUS-mediated antigen release. Contrary to our hypothesis, we find that while these strategies modulate immune abundance, they fail to drive the durable reprogramming necessary to enhance T-FUS-mediated tumor control.

## 2. Materials and Methods

### 2.1. Cell Lines

EMT6 (ATCC, Manassas, VA, USA, Cat. No. CRL-2855) and 4T1 (ATCC, Cat. No. CRL-2539) cell lines were maintained in DMEM (Gibco, Grand Island, NY, USA, Cat. No. 11995073) or RPMI-1640 + L-glutamine (Gibco, Cat. No. 11875-093) supplemented with 10% fetal bovine serum (FBS, Corning, Glendale, AZ, USA, Cat. No. 35-010-CV), respectively. We engineered the EMT6 cell line to stably express ZsGreen fluorescent protein using custom-made lentiviral particles (VectorBuilder, Chicago, IL, USA, VB231002-1295ytz). ZsGreen^+^ EMT6 cells were expanded post-transduction and sorted using a BD Influx cell sorter to enrich for a ZsGreen-positive population. Cells were maintained in the same conditions as the parental cell line. Thawed cells were cultured for at least 1 passage before implantation. All cell lines were tested for mycoplasma contamination prior to cryopreservation.

### 2.2. Animal Maintenance

All animal experiments were conducted according to the University of Virginia guidelines and regulations and approved by the University of Virginia Animal Care and Use Committee. Seven- to nine-week-old female BALB/cJ mice were ordered from The Jackson Laboratory (JAX stock #000651). The right flanks of mice were shaved and 3.0 × 10^5^ EMT6 (parental or ZsG^+^) or 4.0 × 10^5^ 4T1 cells were subcutaneously (s.c.) implanted into the right flank, just above the inguinal lymph nodes, of mice through a 25G × 1 ½ in needle (BD PrecisionGlide Needle, Franklin Lakes, NJ, USA, Cat. No. 305127). Mice were housed on a 12 h/12 h light/dark cycle and ad libitum access to food. Tumor outgrowth was monitored by digital caliper measurements on days 7, 10, and 14 after implantation and every day after treatment initiation. Tumor volume was calculated using the following equation: volume = (length × width^2^)/2. Mice were randomized into groups on the day of treatment to ensure tumor volume means were the same across experimental groups.

### 2.3. Chemotherapies

In total, 1.2 mg/mouse of Gemcitabine (GEM; Hospira, Kalamazoo, MI, USA) and 0.2 mg/mouse of Doxorubicin Hydrochloride (DOX; Fresenius Kabi, Lake Zurich, IL, USA) were diluted in 0.9% saline and administered intraperitoneally (i.p.) on day 14 following tumor inoculation). For flow cytometry studies, a total of 2 chemotherapy doses were given once a week on days 14 and 21 after tumor implantation. Mice that did not receive chemotherapy received an i.p. injection of 500 μL of sterile 0.9% saline. GEM and DOX doses were based on the existing literature [42,43,44].

### 2.4. Depleting Antibodies

In total, 25 µg of anti-Ly6G (1A8 clone; BioXCell, Lebanon, NH, USA, Cat. No. BE0075-1), anti-GR1(RB6-8C5 clone; BioXCell, Cat. No. BE0075), anti-CCR2 (MC-21 clone; provided by Matthias Mack, Regensburg, Germany), or rat isotype controls; IgG2A (2A3 clone; BioXCell, Cat. No. BE0089) and IgG2b (LTF-2 clone; BioXCell, Cat. No. BE0090) were diluted in sterile 0.9% saline and administered i.p. 14 days following tumor inoculation, 2 h before T-FUS to allow the antibody to circulate. Subsequent doses were administered every 2 days for a total of three doses. For flow cytometry studies comparing the different antibodies, only one dose was given on day 12.

### 2.5. Toll-like Receptor (TLR) Agonism

Class B CpG (CpG; 25, 50, or 100 μg/mouse in 100 μL volume; InVivoGen, San Diego, CA, USA, Cat. No. tlrl-1826) and polyinosinic:polycytidylic acid stabilized with poly-L-lysine and carboxymethylcellulose (PolyICLC; 5, 15, or 30 μg/mouse in 100 μL volume; Oncovir, Inc., Washington, DC, USA, Hiltonol^®^) were diluted in 100 µL of limulus amebocyte lysate (LAL) water and administered i.p. at the same time as anti-GR1 (14 days post-tumor inoculation and 2 h before T-FUS).

### 2.6. Flow Cytometry

For tumor analysis, tumors were excised and enzymatically digested for 1 h at 37 °C in RMPI medium supplemented with FBS, 20 U/mL Type I Collagenase (Gibco; Grand Island, NY, USA, Cat. No. 17018029) and 0.1 mg/mL DNase I (Roche; Branchburg, NJ, USA, Cat. No. 10104159001). Following digest, tumors were manually homogenized (Wheaton; Ottawa, ON, CAN Tenbroeck NC, USA, Cat. No. 57-103) and filtered through 100 µm nylon mesh (Genesee Scientific, El Cajon, CA, USA, Cat. No. 57-103) to generate single-cell suspensions. Cells were spun down at 1200 RPM for 5 min and pellets were resuspended in 10 mL 1× PBS. If tumors exceeded 0.5 g, pellets were resuspended in 20 mL 1× PBS and only half the sample was taken for processing. Resuspended tumor samples were underlaid with room temperature 10 mL Lympholyte (Cedarlane Labs; Burlington, ON, Canada, Cat. No. CL5035) and centrifuged at 1000× *g* for 20 min with no acceleration or brake. All layers above the tumor pellet were collected, transferred to a clean 50 mL conical tube, and brought to volume with 1× PBS. Samples were centrifuged again at 800× *g* for 10 min to pellet immune cells. These cells were transferred to a 96 well V-bottom plate for flow staining.

Lymph nodes and spleens were manually homogenized, filtered through 100 µm nylon mesh, and spun down at 1200 RPM for 5 min. Lymph node pellets were immediately transferred to a 96 well V-bottom plate for staining. Spleens pellets were resuspended in 2 mL of Red Blood Cell (RBC) lysis buffer (eBioscience; Middletown, VA, USA, Cat. No. 00-4333-57) for 2 min. After 2 min, RBC lysis buffer was quenched by adding 5 mL of media containing FBS. Samples were spun again at 1200 RPM for 5 min and pellets were transferred to a 96 well V-bottom plate for staining.

For blood analysis, mice were bled via tail veins during outgrowth or retro-orbitally at endpoint. Samples were pelleted, resuspended in 2 mL of RBC lysis buffer for 5 min, quenched with media containing FBS, and centrifuged at 1200 RPM for 5 min. Pellets were transferred to a 96-well V-bottom plate for staining. For chemotherapy studies, blood was taken 24 and 96 h after the first dose and 24 h after the second dose. For depleting antibody experiments, blood was taken 24 h after the first and final doses.

Flow cytometry samples were collected using the Cytek Aurora Borealis (Cytek Biosciences; Fremont, CA, USA) and SpectroFlo v3.0.3 software (Cytek Biosciences). Data were analyzed using FlowJo 10 software (FlowJo, LLC, Ashland, OR, USA). All gating strategies can be found in Figures S1, S4, S6, S7, S9, S12, S16, S17, S19 and S20.

### 2.7. Thermal Ablative Focused Ultrasound

Mice underwent sham or T-FUS treatment 14 days post-tumor inoculation. On treatment day, mice were anesthetized with an i.p. injection of ketamine hydrochloride (20 mg/mL; Zoetis, Parsippany, NJ, USA) and dexmedetomidine hydrochloride (0.05 mg/mL; Dechra, Boston, MA, USA) in sterilized 0.9% saline (Hospira, Cat. No. PAA128035). Anesthesia was reversed with a i.p. injection of atipamezole hydrochloride (Revertidine, Modern Veterinary Therapeutics, Miami, FL, USA) treatment immediately following ablation. Right flanks of mice were shaved to remove regrown hair following tumor implantation and to minimize interference with the FUS treatment. Tumors were sonicated with a custom preclinical FUS system with 4 high power 3.78 MHz therapeutic transducers (SU-102, Sonic Concepts, Bothell, WA, USA) aligned to a single focal point with an active aperture of 66 mm and a central imaging transducer (MS200, FUJIFILM Visualsonics, Toronto, ON, Canada) for treatment guidance as described previously [45]. T-FUS was applied with a 200 W acoustic amplifier (Electronics & Innovation, Rochester, NY, USA, 1020L) driven by an arbitrary function generator (Tektronix, Beaverton, OR, USA, AFG3022C) with treatment with the transducer operated in continuous wave mode at 18 W power for 15 s per point, at 1.5 mm intervals in the X and Y directions, with treatment planes 2 mm apart.

### 2.8. Statistical Anaylsis

Statistical analyses were performed in GraphPad Prism 9 (GraphPad Software). Kaplan–Meier analysis was used to evaluate mouse survival, and statistical significance was determined using the log-rank (Mantel–Cox) test. ROUT outliers’ analysis with Q = 1.0% was performed on all flow cytometry datasets. For flow cytometry summary data collected across multiple timepoints, a full-model two-way analysis of variance (ANOVA) was performed, followed by Dunnett’s post hoc test for multiple comparisons to the control mean. For comparisons involving more than two at a single timepoint, a one-way ANOVA was performed with either Dunnett’s or Tukey’s post hoc tests, as indicated in the figure legends. All figures are presented as mean ± standard deviation (SD). *p*-values and significance are indicated in the corresponding figure legends. Absence of a reported *p*-value denotes a non-significant result.

## 3. Results

### 3.1. Establishing Intratumoral Effects of Myeloid Depletion Strategies

#### 3.1.1. Systemic Myeloablation by GEM and DOX Fail to Modulate Myeloid Cell Niches in the TME

We previously reported that combining GEM with T-FUS in the 4T1 model results in T cell-dependent tumor control; however, these responses are not durable, and mice ultimately succumb to disease [41]. More recent data from our laboratory indicate that GEM does not reduce myeloid cell abundance within the 4T1 TME, despite its systemic myeloablative activity [42]. Moreover, GEM treatment significantly reduced the number of infiltrating T cells, B cells, and dendritic cells [42]. Therefore, we sought to determine whether an alternative chemotherapy capable of inducing immunogenic cell death (ICD) could more effectively and durably ablate isMCs from the TME. DOX was selected because, unlike GEM, which is generally used as a later-line therapy for metastatic BrCa [46,47,48], DOX is a clinically established first-line anthracycline for BrCa reported to possess myeloablative potential [49]. In addition to its clinical relevance, DOX is a well-characterized inducer of ICD [50,51,52,53]—a form of regulated cell death capable of activating the adaptive immune system. Although DOX carries a less favorable toxicity profile than GEM [54,55], its ability to stimulate immunogenic tumor cell death provided a strong rationale for evaluating whether it could improve the durability of tumor control in combination with our therapeutic approach.

Mice bearing established EMT6 tumors received one intraperitoneal (i.p.) dose of 1.2 mg GEM or 0.2 mg DOX on day 14 post-tumor implantation, with some tumor-bearing mice receiving a second dose one week later. Consistent with our previous studies administering GEM in the 4T1 model, we found that GEM treatment actually increased intratumoral neutrophil number and proportion 24 h after the initial injection when compared to saline controls (Figure S2(Ai,Aii)) [42]. Ninety-six hours after the initial dose, neutrophil number and proportion was reduced, but this reduction was not sustained with a second dose. DOX induced a transient decrease in neutrophil numbers at 24 h, but this effect was also lost by 96 h (Figure S2(Ai,Aii)). Neither chemotherapy ablated either Ly6C^+^ or Ly6C^hi^ monocytes at any timepoint (Figure S2(Ci,Cii,Di,Dii)), but both significantly reduced macrophage numbers 24 h after the first dose (Figure S2(Bi)). Furthermore, neither therapy increased the number of infiltrating cDCs, T cells, or B cells 24 h after the first chemotherapeutic dose; in fact, there was a timing specific decrease in some of these populations following either chemotherapy (Figure S3). DOX is reported to induce ICD, typically measured by calreticulin exposure or HMGB1 expression [50,51,52,53]. Although not measured here, we used CD86 expression by DC in the tumor-draining lymph nodes (tdLN) as an indirect readout of ICD [56,57]. We found that GEM, but not DOX, increased the proportion of activated CD86^hi^ cDCs at the most acute timepoint, but neither chemotherapy increased the absolute number of activated cDC1s or cDC2s in the tdLN (Figure S5). Overall, these data indicate that standard myeloablative chemotherapies fail to substantially and durably remodel intratumoral myeloid populations.

#### 3.1.2. Antibody-Based Targeting of Myeloid Subsets Reveals Limits and Opportunities

**Figure 1:**
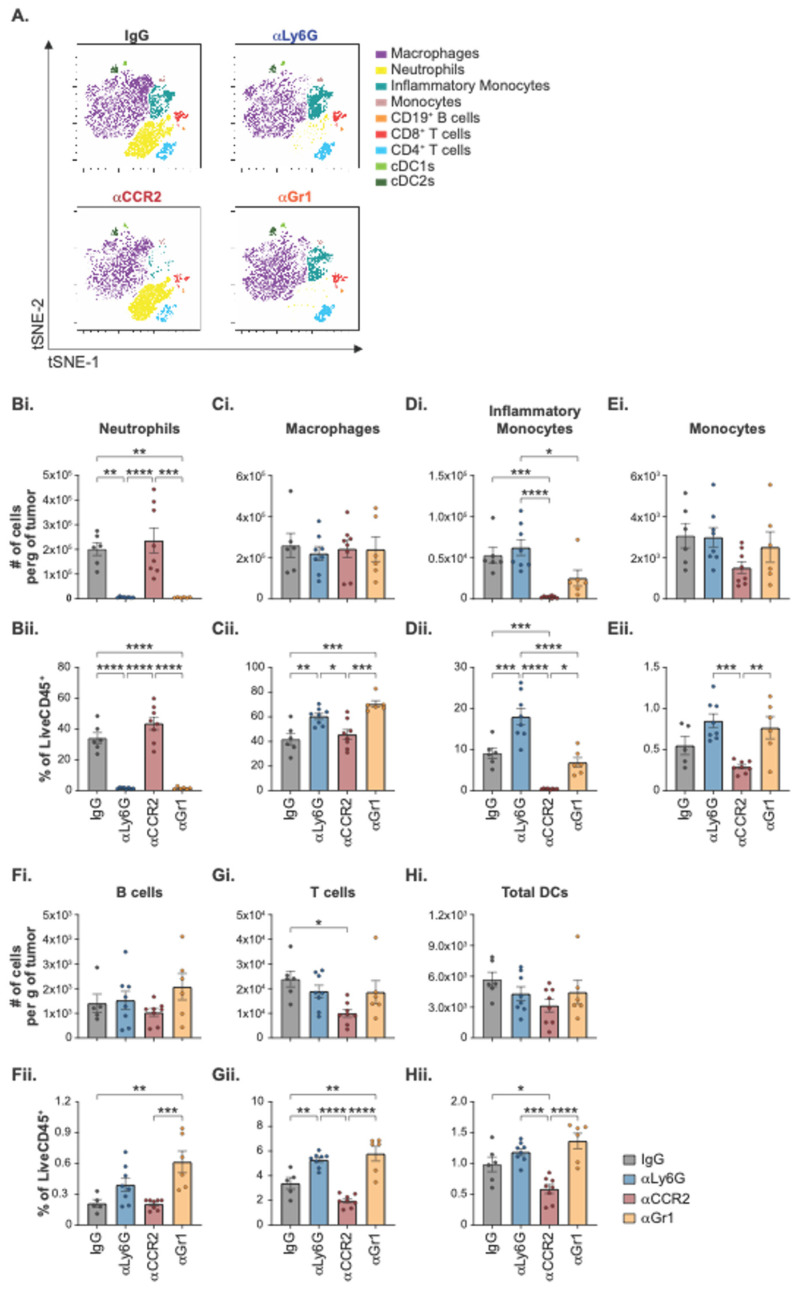
Antibody-mediated myeloid depletion reshapes the EMT6 TME. 300k parental EMT6 cells were inoculated in the right flanks of BALB/c mice. Mice were injected with 25 μg of αLy6G, αGr1, αCCR2, or saline i.p. on d 12 and 14 post-inoculation. Tumors were excised 24 h after final dose on d15. (**A**). tSNE dimensionality reduction on LiveCD45^+^ cells in the TME. (**Bi**,**Bii**). Changes in neutrophil number (**Bi**) and proportion (**Bii**). (**Ci**,**Cii**). Changes in macrophage number (**Ci**) and proportion (**Cii**). (**Di**,**Dii**). Changes in inflammatory monocyte number (**Di**) and proportion (**Dii**). (**Ei**,**Eii**). Changes in monocyte number (**Ei**) and proportion (**Eii**). (**Fi**,**Fii**). Changes in B cell number (**Fi**) and proportion (**Fii**). (**Gi**,**Gii**). Changes in T cell number (**Gi**) and proportion (**Gii**). (**Hi**,**Hii**). Changes in total cDC number (**Hi**) and proportion (**Hii**). (*n* = 8) (one-way ANOVA followed by Tukey’s post hoc test for multiple comparisons: * *p* < 0.05, ** *p* < 0.01, *** *p* < 0.001, **** *p* < 0.0001; ROUT Outliers analysis with Q = 1.0%). All points represent mean ± SD.

To evaluate the ability of myeloid-targeting antibodies to reduce isMC presence in tumors, we compared anti-Ly6G (clone 1A8), which specifically recognizes Ly6G and therefore selectively depletes neutrophils [58,59] and anti-Gr1 (clone RB-8C5), which binds both Ly6G and Ly6C and consequently targets neutrophils as well as Ly6C-expressing monocytes, macrophages, and some T cell subsets [59,60]. Both antibodies effectively depleted intratumoral and systemic neutrophil populations, whereas only anti-Gr1 reduced intratumoral and systemic monocytes—most notably the Ly6C^hi^ inflammatory subset (Figure 1 and Figure S8). These effects were transient and accompanied by an unexpected decrease in circulating T cells (Figure S8(Gi,Gii)). The same low-dose anti-Gr1 and anti-Ly6G regimens were previously evaluated in mice bearing established 4T1 tumors but proved lethal, even after titrating the doses and revising the treatment regimen. Therefore, all subsequent myeloid depletion experiments were conducted using the EMT6 tumor model.

We also considered whether targeting CCR2, a chemokine receptor that directs the migration of monocytes to tumors or sites of tissue damage [61,62,63], would result in selective reduction in immunosuppressive monocyte-derived myeloid cells. Like anti-Gr1 treatment, anti-CCR2 antibody therapy led to a significant reduction in inflammatory monocytes in the tumor and systemically (Figure 1 and Figure S8), but had a greater impact on intratumoral CD3^+^ cells. In contrast, although anti-Gr1 reduced circulating T cells, it did not measurably deplete T cells within the TME. For this reason, we elected to use anti-Gr1 for further studies with T-FUS as this strategy enables more selective removal of multiple myeloid subsets while minimizing unintended effects on T cells.

### 3.2. Limited Myeloid Reprogramming in the TME Despite Robust cDC Activation in the tdLN

#### 3.2.1. TLR Agonism Fails to Reprogram Suppressive Myeloid Populations Within the TME

We hypothesized that TLR agonism would functionally reprogram isMCs in established EMT6 tumors leading to reduced arginase 1 (Arg1)-associated immunosuppression [64,65,66,67,68,69,70]. We performed initial dose-ranging assays in which tumor-bearing mice received either 20 μg, 50 μg, or 100 μg CpG-B (TLR9 agonist) or 5 μg, 15 μg, or 30 μg poly-ICLC (TLR3 agonist) i.p., and myeloid phenotypes were assessed in the tumor 24 h later. We observed no reduction in the number of Arg1^+^SIRPα^+^ neutrophils across all CpG-B doses tested; however, total Arg1 GMF decreased (Figure S10(Ai–B)). These data indicate that CpG may not reprogram Arg1^+^SIRPα^+^ neutrophils but instead expand Arg1^-^ neutrophil subsets or promote arginase release through degranulation [71,72], thereby lowering total Arg1 detected across the entire neutrophil population (Figure S10(Aiii)). Notably, in these small-cohort studies, low-dose CpG-B elicited an increase in Arg1 GMF within the Arg1^+^ neutrophil gate, indicating selective upregulation or retention of arginase in a subset of neutrophils (Figure S10(Aiv)). In contrast, poly-ICLC treatment led to a dose-dependent reduction in the number of Arg1^+^ neutrophils with similar trends in Arg1 GMF both within Arg1^+^ neutrophils and across the total neutrophil compartment (Figure S11(Ai,B)).

Because functional reprogramming in neutrophils may be difficult to assess due to neutrophil activation and degranulation, we also determined whether TLR agonism could reprogram other suppressive myeloid populations within EMT6 tumors. While acknowledging that binary classifications of highly plastic cells cannot fully capture the dynamic programming of macrophages, we evaluated Arg1 and MHCII expression as a proxy to define pro- (Arg1^−^MHCII^+^) and anti-inflammatory (Arg1^+^MHCII^−^) states within these preliminary dose-finding analyses. Neither CpG-B nor poly-ICLC increased the ratio of Arg1^−^MHCII^+^ to Arg1^+^MHCII^−^ inflammatory monocytes or macrophages in EMT6 (Figure S10(Ci)) or 4T1 tumors (Figure S12(Ci)), with the exception of a modest shift at the lowest CpG-B dose in macrophages (Figure S10(Ei–F)). Despite favorable skewing, this phenotypic change was not accompanied by reductions in Arg1 GMF in either Arg1^+^ or total macrophages (Figure S10(Eii,Eiii)) or meaningful increases in inducible nitric oxide synthase (iNOS) production by iNOS^+^ neutrophils, inflammatory monocytes, or macrophages at this low dose (Figure S10(Gi–Giii)). Importantly, the pro-/anti-inflammatory ratio still favored the Arg1^+^MHCII^−^ phenotype which may explain why Arg1 production does not differ from control tumors in either Arg1^+^ cells or total inflammatory monocytes and macrophage compartments (Figure S10(Ci,Ei)). Instead, both TLR agonists produced effects more consistent with ablative strategies, characterized by broad reductions in total immune cell abundance within the TME and systemically. Collectively, these initial findings suggest that, at the doses and timepoints evaluated, TLR agonism alters immune cell abundance more readily than it induces functional reprogramming of suppressive myeloid populations in EMT6 and 4T1 tumors.

Within this pilot study, we also found that the highest dose of CpG elicited significant expansion of total cDCs (Figure S14(Ci,Cii)) and activation (CD86^hi^) of both cDC1 and cDC2s in tdLN in EMT6 (Figure S15(Ai–C)), whereas none of the poly-ICLC doses produced comparable increases (Figures S14(Ciii,Civ) and S16(Ai–C)). An increase in CD103 expression by tdLN cDCs was consistent with the notion that CpG promotes migration from the periphery to the tdLN (Figure S15(Di,Dii)), which was not observed with poly-ICLC (Figure S16(Di,Dii)); however, the lack of statistical significance may reflect the limited cohort size in this experiment. These findings were also recapitulated in 4T1-bearing mice treated with 100 μg of CpG or 15 μg of Poly-ICLC, showing superior cDC activation and migration following high-dose CpG (Figure S17). Given the combined effects of 100 μg CpG, we elected to use this dose for subsequent studies.

#### 3.2.2. Intratumoral Delivery Fails to Overcome CpG-Induced Reprogramming Deficits

**Figure 2:**
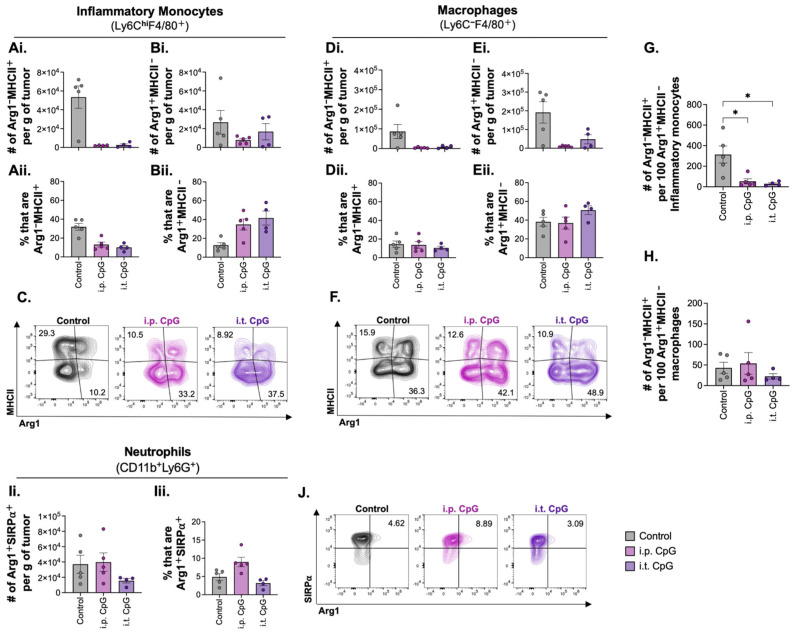
Neither i.p. nor i.t. CpG-B administration reverses myeloid programming to a pro-inflammatory state. 300k ZsG^+^ EMT6 cells were inoculated in the right flanks of BALB/c mice. Mice were injected with CpG (100 μg) i.p. or i.t. on day 14 post-inoculation. Tumors were excised 24 h post-injection. (**Ai**,**Aii**). Changes in Arg1^−^MHCII^+^ inflammatory monocyte number (**Ai**) and proportion (**Aii**). (**Bi**,**Bii**). Changes in Arg1^+^MHCII^−^ inflammatory monocyte number (**Bi**) and proportion (**Bii**). (**C**). Representative flow plots for A-B. (**Di**,**Dii**). Changes in Arg1^−^MHCII^+^ macrophage number (**Di**) and proportion (**Dii**). (**Ei**,**Eii**). Changes in Arg1^+^MHCII^−^ macrophage number (**Ei**) and proportion (**Eii**). (**F**). Representative flow plots for D-E. (**G**) Ratio of Arg1^−^MHCII^+^ normalized to 100 Arg1^+^MHCII^−^ inflammatory monocytes. (**H**) Ratio of Arg1^−^MHCII^+^ normalized to 100 Arg1^+^MHCII^−^ macrophages. (**Ii**,**Iii**) Changes in the number of Arg1^+^SIRPα^+^ neutrophils (**Ii**) and the proportion (**Iii**). (**J**). Representative flow plots for G. (*n* = 4–5) (one-way ANOVA followed by Tukey’s post hoc test for multiple comparisons: * *p* < 0.05; ROUT Outliers analysis with Q = 1.0%). All points represent mean ± SD.

Having selected the highest dose of CpG from the pilot studies, we determined if the delivery route could influence efficacy in the EMT6 model. We compared the ability of i.p. and intratumoral (i.t.) delivery of CpG (100 μg) to reprogram myeloid cells while preserving the robust cDC response described above (Figure S15). Neither route drove pro-inflammatory reprogramming of isMCs (Figure 2). Furthermore, despite the accumulation of CD103^+^ cDCs described above, direct tracking of ZsGreen (ZsG)^+^ tumor antigen revealed no significant increases in antigen acquisition by cDCs in the tdLN with either route (Figure S18). This suggests CpG-B fails to mobilize tumor-antigen-bearing cDCs effectively, regardless of administration route.

### 3.3. Combining Myeloid Manipulating Strategies with T-FUS Provided No Additional Tumor Control

**Figure 3:**
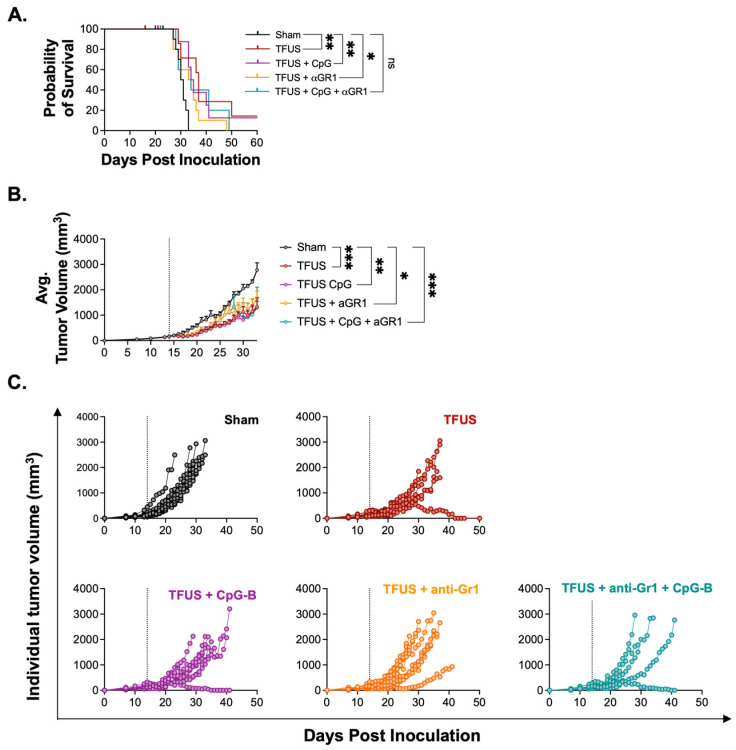
T-FUS remains the principal driver of tumor control. 300k parental EMT6 cells were inoculated in the right flanks of BALB/c mice. Mice were treated with T-FUS on day 14 post-inoculation. Tumors were excised 24 h post-treatment. (**A**). Kaplan–Meier curve depicting overall survival (significance assessed by log-rank Mantel–Cox test: * *p* < 0.05, ** *p* < 0.01). (**B**). Averaged tumor volume over 33 days (until significant dropout impacted averages). Dashed line represents T-FUS treatment day (d14). One-way ANOVA followed by Tukey’s post hoc test for multiple comparisons: * *p* < 0.05, ** *p* < 0.01, *** *p* < 0.001; ROUT Outliers analysis with Q = 1.0%). All points represent mean ± SD. (**C**) Individual tumor volumes. (*n* = 7).

Given the ability of T-FUS to promote tumor antigen release and availability, we tested whether its addition to anti-Gr1 and high-dose CpG would cooperatively increase durable tumor control. As expected, all T-FUS-containing treatment groups exhibited significant tumor control as measured by tumor volume. Most T-FUS-containing treatment groups also exhibited extended survival compared to sham controls. Notably, the triple-combination group was the only T-FUS-containing cohort that failed to demonstrate a significant survival advantage, likely due to treatment-associated wound complications that met experimental endpoint guidelines. Even so, these data indicate that CpG and anti-Gr1 conferred no additional therapeutic benefit in any combination (Figure 3).

**Figure 4:**
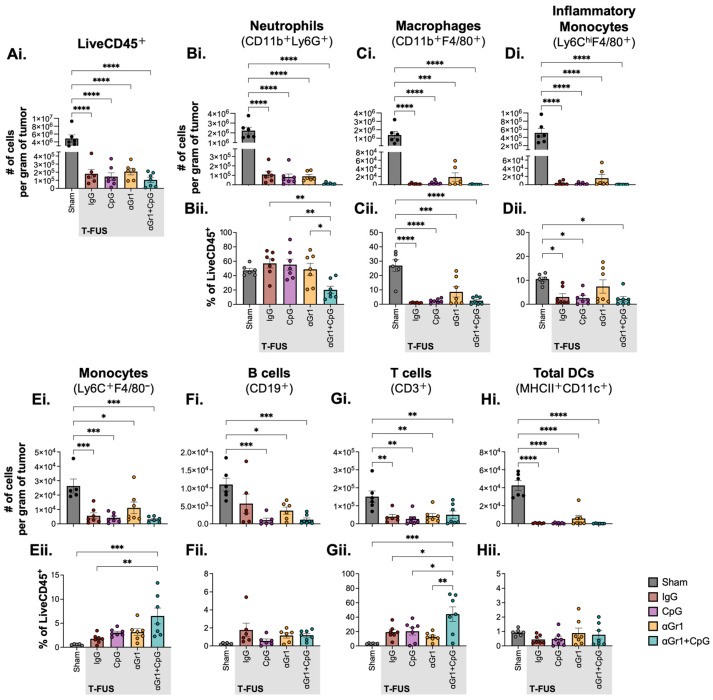
T-FUS drives robust ablation of several immune cell subsets. 300k ZsG^+^ EMT6 were inoculated in the right flanks of BALB/c mice. Mice were treated with T-FUS on day 14 post-inoculation. Tumors were excised 72 h post treatment. (**Ai**). Changes in total Live-CD45^+^ cell numbers per gram of tumor. (**Bi**,**Bii**). Changes in neutrophil number (**Bi**) and proportion (**Bii**). (**Ci**,**Cii**). Changes in macrophage number (**Ci**) and proportion (**Cii**). (**Di**,**Dii**). Changes in inflammatory monocyte number (**Di**) and proportion (**Dii**). (**Ei**,**Eii**). Changes in monocyte number (**Ei**) and proportion (**Eii**). (**Fi**,**Fii**). Changes in B cell number (**Fi**) and proportion (**Fii**). (**Gi**,**Gii**). Changes in T cell number (**Gi**) and proportion (**Gii**). (**Hi**,**Hii**). Changes in total cDC number (**Hi**) and proportion (**Hii**). (*n* = 7) (one-way ANOVA followed by Tukey’s post hoc test for multiple comparisons: * *p* < 0.05, ** *p* < 0.01, *** *p* < 0.001, **** *p* < 0.0001; ROUT Outliers analysis with Q = 1.0%). All points represent mean ± SD.

To understand whether the combination treatments resulted in significant acute immunologic remodeling of the tumor microenvironment, we profiled tumors after treatment. T-FUS resulted in robust tumor ablation irrespective of accompanying immunologic therapeutics, accompanied by significant reductions across all assessed cell populations (Figure 4). Notably, within T-FUS treated tumors, the combination of CpG and anti-GR1, but not anti-GR1 alone, decreased the relative proportion of neutrophils, while the combination increased the proportion of CD3^+^ T cells (Figure 4Bi,Bii,Ei,Eii,Gi,Gii).

**Figure 5:**
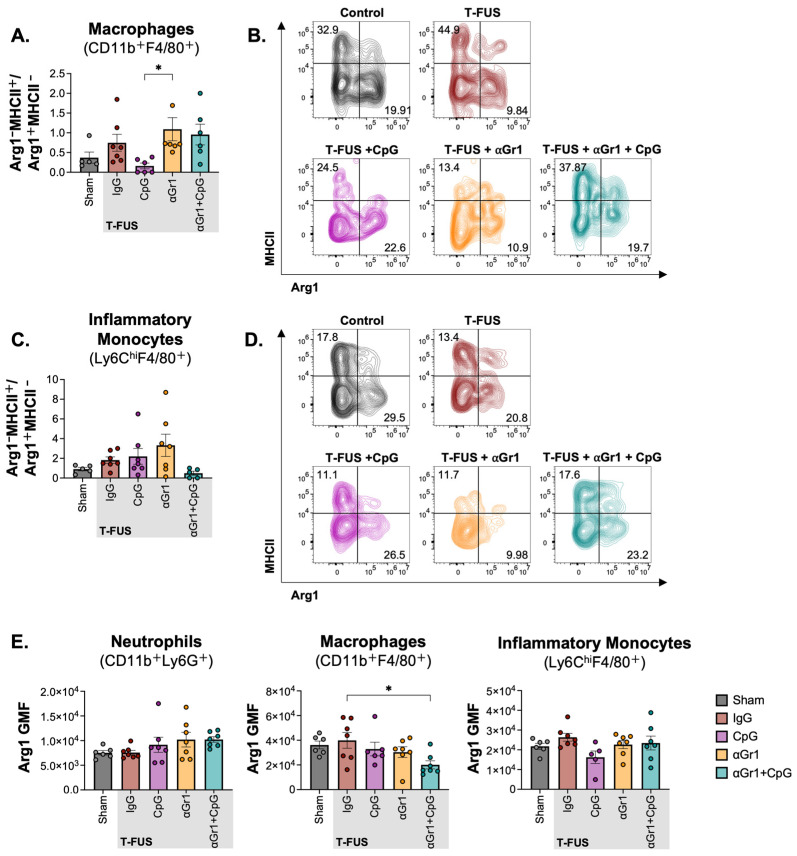
Dual- and triple-combination strategies are inefficient at myeloid reprogramming. 300k ZsG^+^ EMT6 cells were inoculated in the right flanks of BALB/c mice. Mice were treated with T-FUS on day 14 post-inoculation. Tumors were excised 72 h post-treatment. (**A**). Changes in the ratio of Arg1^−^MHCII^+^ per Arg1^+^MHCII^−^ macrophages. (**B**). Representative flow plots for Arg1 and MHCII expression on macrophages by each treatment group. (**C**). Changes in the ratio of Arg1^−^MHCII^+^ per Arg1^+^MHCII^−^ inflammatory monocytes. (**D**). Representative flow plots for Arg1 and MHCII expression on inflammatory monocytes by each treatment group. (**E**). Changes in Arg1 GMF by total neutrophils, macrophages, and inflammatory monocytes (*n* = 7) (one-way ANOVA followed by Tukey’s post hoc test for multiple comparisons: * *p* < 0.05; ROUT Outliers analysis with Q = 1.0%). All points represent mean ± SD. Representative flow plots were picked from down sampled data concatenated on the LiveCD45^+^ gate.

**Figure 6:**
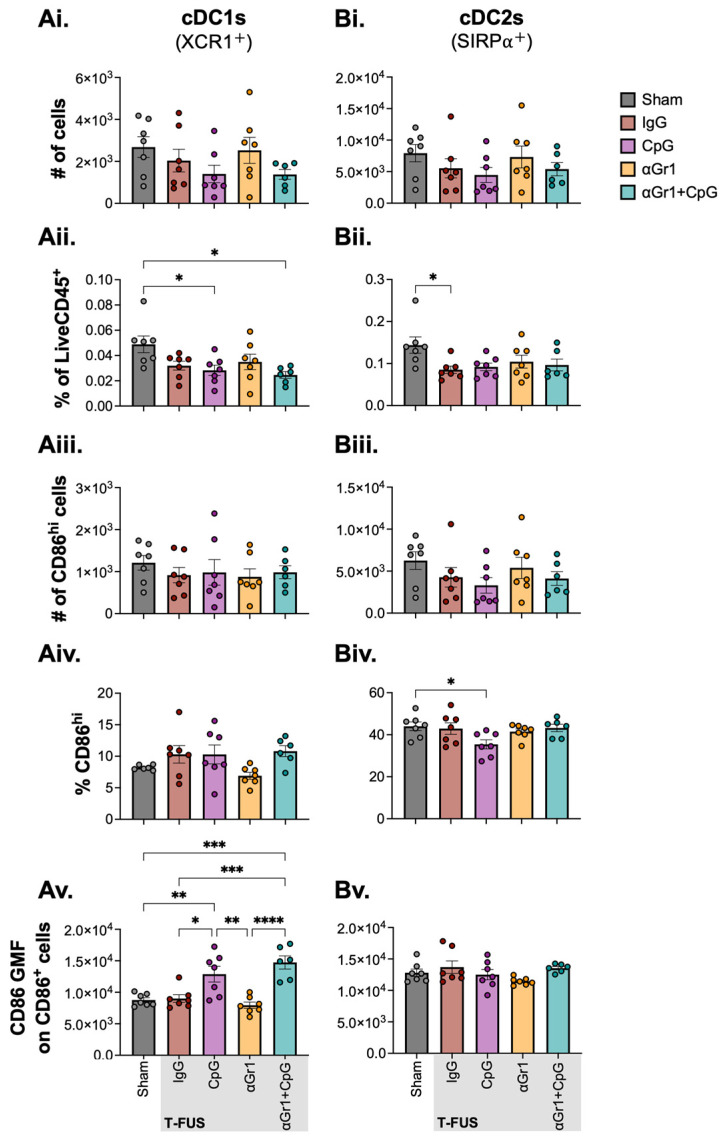
Neither dual- or triple-combination therapy increases cDC numbers and activation. 300k ZsG^+^ EMT6 were inoculated in the right flanks of BALB/c mice. Mice were treated with T-FUS on day 14 post inoculation. TdLN were excised 72 h post treatment. (**Ai**–**Av**). Changes in cDC1 number (**Ai**) and proportion (**Aii**). Changes in CD86^hi^ cDC1 number (**Aiii**) and proportion (**Aiv**). Changes in CD86 GMF on CD86^+^ cDC1s (**Av**). (**Bi**–**Bv**). Changes in cDC2 number (**Bi**) and proportion (**Bii**). Changes in CD86^hi^ cDC2 number (**Biii**) and proportion (**Biv**). Changes in CD86 GMF on CD86^+^ cDC2s (**Bv**) (*n* = 7) (one-way ANOVA followed by Tukey’s post hoc test for multiple comparisons: * *p* < 0.05, ** *p* < 0.01, *** *p* < 0.001, **** *p* < 0.0001; ROUT Outliers analysis with Q = 1.0%). All points represent mean ± SD.

We also assessed myeloid functional states by quantifying the ratio of pro-inflammatory to anti-inflammatory macrophages and inflammatory monocytes, as well as Arg1 GMF across neutrophils, macrophages, and inflammatory monocytes. Although certain combination strategies produced modest shifts in the pro- to anti-inflammatory ratios, these changes were not biologically meaningful with the balance remaining closer to one-to-one in most cases (Figure 5A–D). This interpretation was supported by finding that Arg1 expression remained largely unchanged across all subsets assessed, indicating that neither T-FUS alone nor any of the combinatorial strategies alleviated Arg1 expression relative to sham controls. These findings recapitulate the limited reprogramming observed with CpG-B alone at 24 h post-injection (Figure 2 and Figure 5). Moreover, CpG-driven cDC activation, which was readily detectable at earlier timepoints, was modest when combined with T-FUS at this later timepoint, suggesting that CpG-B-mediated cDC activation is transient or is abrogated by T-FUS ablation (Figure 6(Ai–Av,Biii–Bv)). Consistent with this, we did not observe an increase in ZsG in the tdLN. Together, these data indicate that T-FUS—either alone or combined with CpG and/or Gr1 depletion—fails to induce durable myeloid reprogramming, despite producing strong ablative effects and altering overall intratumoral immune composition. Given the relative inflation of T cells and reduction in neutrophils, it is unclear why the triple-combination strategy did not provide superior tumor control or improve survival relative to dual-combination therapies or sham treatment.

## 4. Discussion

In this study, we systematically interrogated multiple strategies to relieve myeloid-mediated immunosuppression in the EMT6 TNBC tumor microenvironment. Contrary to our hypothesis—based on prior work demonstrating a T cell-dependent therapeutic advantage when GEM was combined with T-FUS [41] and our previous data suggesting GEM is suboptimal in reducing isMCs in the TME [42]—none of the interventions durably depleted or reliably reprogrammed isMCs within established EMT6 tumors, nor did they enhance the therapeutic effect of T-FUS.

Consistent with our previous work in the 4T1 model, GEM did not sustainably deplete intratumoral isMCs despite causing systemic myeloablation (Figure S2). Unexpectedly, DOX—despite its well-described capacity to induce ICD [50,51,52,53]—likewise failed to durably reduce isMC abundance (Figure S2). DOX induced a transient reduction in neutrophils but this was not accompanied by increased lymphocyte infiltration or cDC activation (Figures S2 and S3). The observed reduction in macrophages achieved by both chemotherapies, despite their terminal differentiation and limited proliferative capacity (Figure S2), may result from a chemotherapy-induced loss of tumor-derived macrophage colony-stimulating factor (MCSF), a cytokine critical for macrophages survival and maintenance [73,74,75,76]. While anthracyclines like DOX are used as prototypic ICD-inducing agents [50,51,52,53,77,78], we observed no corresponding increase in cDC activation in the tdLN, suggesting that ICD-associated signals may be insufficient, transient, or uncoupled from induction of costimulatory molecules in this setting.

Similar to GEM and DOX, targeting Ly6G or Gr1 effectively depleted circulating neutrophils, with anti-Gr1 additionally reducing monocyte and inflammatory monocyte populations (Figure S8(A–Eii)) [41]. However, both antibodies were associated with unintended reductions in circulating CD8α^+^ and CD4^+^ T cells (Figure S8(H–Jii)). These depletion profiles were largely recapitulated within tumors except infiltrating T cells were spared by Ly6G and Gr1 targeting antibodies (Figures S2 and S3). Although the proportion of infiltrating T cells increased, the absolute numbers stayed the same suggesting that the reductions seen in the blood were not because of T cell trafficking to the tumor. Further, the increase in the proportion of T cells is likely a function of a reduction in other immune cells subsets rather than an expansion of T cells, though this needs to be confirmed experimentally.

CCR2 is a chemokine receptor that directs the migration of monocytes to tumors or sites of tissue damage. Its ligand, CCL2 [79], can serve as a poor prognostic indicator in BrCa as it is frequently expressed in breast tumors, correlates with increased monocyte/macrophage recruitment, and is associated with advanced disease [63,80,81]. Anti-CCR2 antibody selectively depleted monocytes and inflammatory monocytes yet also reduced intratumoral T cell abundance (Figure S8). Together, these findings highlight a fundamental challenge in myeloid directed therapy: selective depletion is difficult to obtain, and effects can be transient which is consistent with compensatory myelopoiesis. It is tempting to speculate that additional rounds of depletion would lead to more durable reductions; however, the development of anti-antibody responses temper this approach.

We hypothesized that TLR agonism could serve as an alternative to depletion by functionally reprogramming isMCs, thereby alleviating myeloid-mediated immunosuppression without inducing compensatory myelopoiesis. However, across both EMT6 and 4T1 models, TLR agonists failed to induce durable shifts toward pro-inflammatory (Arg1^−^MHCII^+^) myeloid phenotypes. (Figures S10–S12). Instead, both CpG and poly-ICLC acted as ablative agents, causing broad loss of most immune cell subtypes. These findings contrast with reports of TLR-mediated myeloid reprogramming in other tumor models [82,83,84] and suggest that tumor context influences the malleability of innate immune reprogramming.

Despite limited evidence for myeloid cell reprogramming, we did note that, in contrast to DOX, high-dose CpG-B robustly activated and mobilized cDCs to the tdLNs following both i.p. and i.t. delivery (Figure 2), yet failed to enhance tumor antigen presence in the tdLN (Figure S20), pointing to antigen availability as a potential bottleneck. However, the combination therapy neither resulted in an increase in antigen presence in the tdLN nor improve tumor control or survival (Figure 5), despite leading to a relative increase in T cell representation in the treated tumors. Although we could not assess the functional state of the residual intratumoral CD3^+^ T cells due to tissue limitations (and thus cannot exclude the possibility that the myeloid manipulations did lead to increased T cell function), we did evaluate PD-1 and TIM-3 expression on circulating CD8^+^ T cells, quantifying both the frequency and intensity of expression in Sham, CpG, anti-GR1, and anti-GR1+CpG treatment groups (without TFUS). We observed no differences across treatment groups. Interpreting T cell functionality in this context remains challenging given the lack of clearly defined tumor antigens and the scarcity of T cells within treated tumors. Moreover, any impact on survival that might have been achieved by increased frequency and/or function of intratumoral CD3^+^ T cells may have been offset by a high incidence of treatment-associated wound complications, limiting the power of the study. Given the established role of neutrophils in tissue repair [85,86], depleting them may also have compromised wound resolution and survival. Moreover, the addition of a TLR agonist that itself exerts ablative effects may have further exacerbated tissue damage. These observations suggest that the timing and sequencing of these interventions, particularly when used in combination, are a critical consideration [87,88,89].

A constraint on the conclusions generated from these studies presented here is the inherent limitation that accompanies examining alterations in the phenotype and functionality of immune cell populations at specific timepoints post-treatment. We recognize that the generalizability of these observations is limited by the preliminary nature of dose-ranging studies, the potential for suboptimal dosing and particularly the duration of treatments; the extent of tumor lines studied; and that varied forms of poly-ICLC and CpG may have different outcomes than reported here. Thus, while the data presented here (and summarized in Table 1) suggest that the myeloid compartment is resistant to manipulation and that depletion can be accompanied by deleterious side effects, the current data do suggest that our previously reported synergy between GEM and T-FUS [41] may not be mediated through relief of myeloid suppression but rather through direct effects on the tumor or by non-immune stromal cells. In the myeloid-rich EMT6 model, T-FUS acts as a dominant physical ablative modality. Neither myeloid depletion nor innate immune activation is sufficient to convert local tissue damage into more durable tumor control.

## Figures and Tables

**Table 1:** Summary of observed effects on myeloid cells in the two models of TNBC.

Feature/Treatment	EMT6	4T1
GEM Effect	Systemic myeloablation; fails to reduce intratumoral isMCs.	Systemic myeloablation; fails to reduce intratumoral isMCs [42].
DOX Effect	Transient neutrophil reduction; no cDC activation.	No data.
Ab-Mediated Depletion	Tolerated; effective systemic/intratumoral depletion (though transient).	Lethal toxicity; regimens proved fatal even after dose titration.
TLR Agonism (CpG)	Failed to reprogram isMCs; induced broad immune cell loss (ablative).	Failed to reprogram isMCs; induced broad immune cell loss (ablative), Arg1+ status maintained.
TLR Agonism (Poly-ICLC)	Dose-dependent reduction in Arg1+ neutrophils, but no stable phenotypic shift.	No meaningful reprogramming observed.
cDC Mobilization	Robust cDC activation/migration to tdLN with high-dose CpG.	Recapitulated high-dose CpG response (100 μg).
T-FUS Interaction	T-FUS is the dominant driver; no additive benefit from Ab/TLR.	Synergy with GEM reported previously [41].

## Data Availability

The original contributions presented in this study are included in the article/Supplementary Materials. Further inquiries can be directed to the corresponding author.

## Supplementary Materials

The following supporting information can be downloaded at: https://www.mdpi.com/article/10.3390/cells15111035/s1.
