# Supplementary material for: Myeloid Cell Targeting Strategies Show Limited Durable Activity in the Breast Cancer Tumor Microenvironment and Do Not Enhance the Activity of Thermally Ablative Focused Ultrasound
Source: Cells. 2026 Jun 4;15(11):1035. doi: 10.3390/cells15111035 (PMC13256334; doi:10.3390/cells15111035)
Supplement: Supplementary file 1 [file cells-15-01035-s001.zip › cells-4282456-supplementary.pdf]

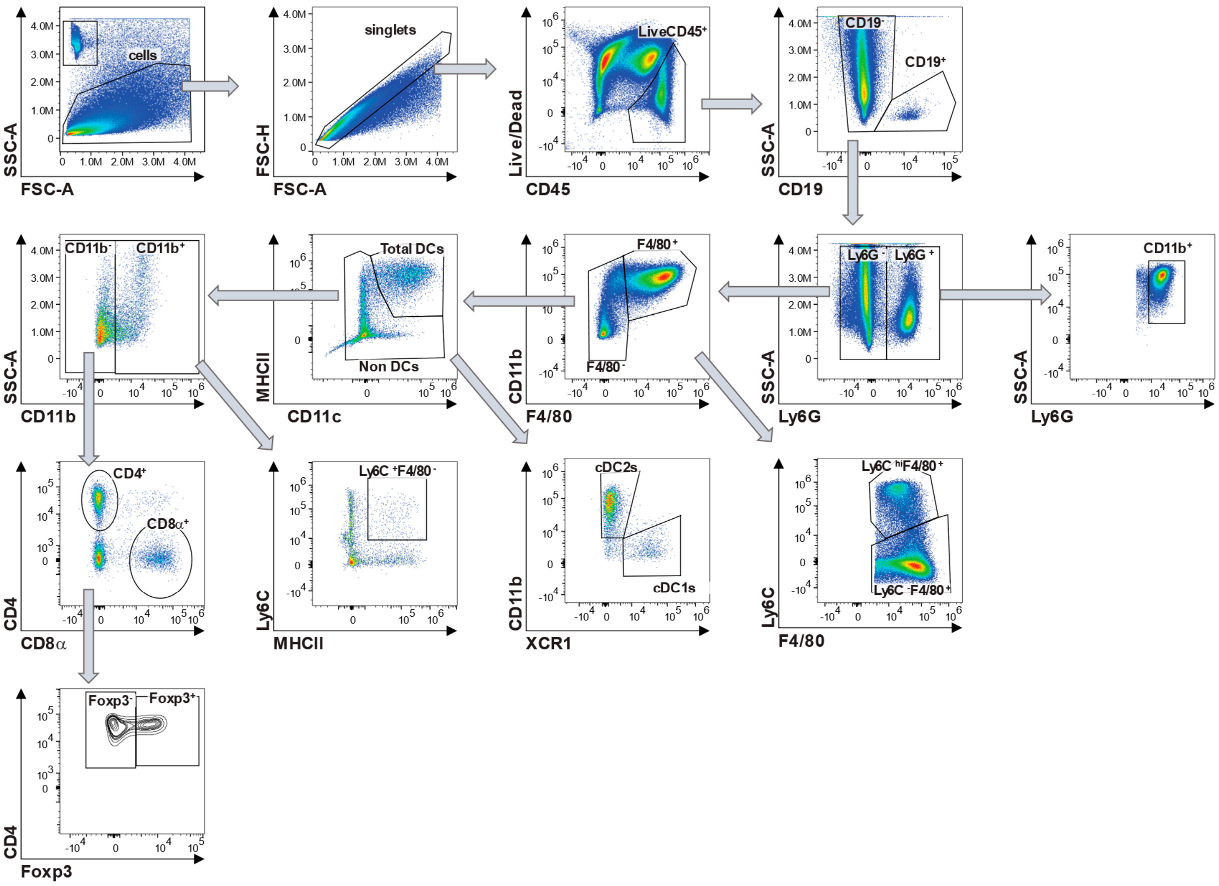

Figure S1. Gating strategy for tumor flow cytometry analysis in Figure S2 & S3. Gating strategy presented for the following immune cell subsets: neutrophils (LiveCD45$^+$CD19$^-$Ly6G$^+$CD11b$^+$); macrophages (LiveCD45$^+$CD19$^-$Ly6G$^-$CD11b$^+$F4/80$^+$Ly6C$^-$); inflammatory monocytes (LiveCD45$^+$CD19$^-$Ly6G$^-$CD11b$^+$F4/80$^+$Ly6C^{hi}); monocytes (LiveCD45$^+$CD19$^-$Ly6G$^-$F4/80$^-$NonDCs CD11b$^+$Ly6C$^+$); dendritic cells (LiveCD45$^+$CD19$^-$Ly6G$^-$F4/80$^-$CD11c$^+$MHCII$^+$); CD8α T cells (LiveCD45$^+$CD19$^-$Ly6G$^-$F4/80$^-$NonDCs CD11b$^-$CD4$^-$CD8α$^+$); CD4 Helper T cells (LiveCD45$^+$CD19$^-$Ly6G$^-$F4/80$^-$NonDCs CD11b$^-$CD4$^+$CD8α$^-$Foxp3$^-$); and B cells (LiveCD45$^+$CD19$^+$).

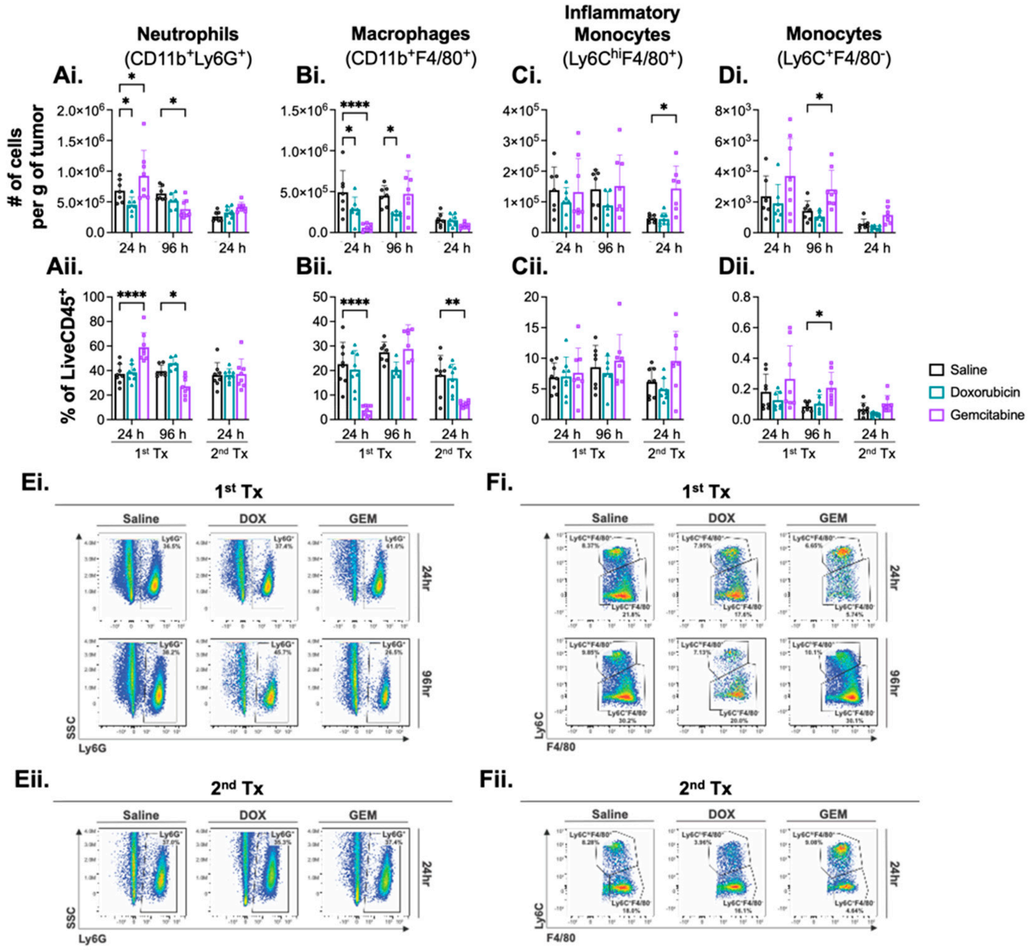

Figure S2. Gemcitabine (GEM) and doxorubicin (DOX) do not act as myeloablative therapies in the EMT6 tumor microenvironment. 300k parental EMT6 cells were inoculated in the right flanks of BALB/c mice. Mice were injected with GEM (1.2mg), DOX (0.2mg) or saline i.p. on day 14 post inoculation. Tumors were excised 24 and 96 h post initial injection and 24 h post second injection 1 week later. (**A**). Changes in neutrophil number (**Ai**) and proportion (**Aii**) 24 and 96 h after the first treatment injection (Tx) and 24 h after the second Tx. (**B**). Changes in macrophage number (**Bi**) and proportion (**Bii**) 24 and 96 h after the first Tx and 24 hr after the second Tx. (**C**). Changes in inflammatory monocyte number (**Ci**) and proportion (**Cii**) 24 and 96 h after the first Tx and 24 h after the second Tx. (**D**). Changes in macrophage number (**Di**) and proportion (**Dii**) 24 and 96 h after the first Tx and 24 h after the second Tx. (**E**) Representative flow plots of neutrophils 24 and 96 h after the 1st Tx (**Ei**) and 24 h after the 2nd Tx (**Eii**). (**F**) Representative flow plots of macrophages and inflammatory monocytes 24 and 96 h after the 1st Tx (**Fi**) and 24 h after the 2nd Tx (**Fii**). (n=8) (two-way ANOVA followed by Dunnett's post hoc test for multiple comparison to the control group: * $p < 0.05$, ** $p < 0.01$, **** $p < 0.0001$; ROUT Outliers analysis with Q = 1.0%). All points represent mean ± SD.

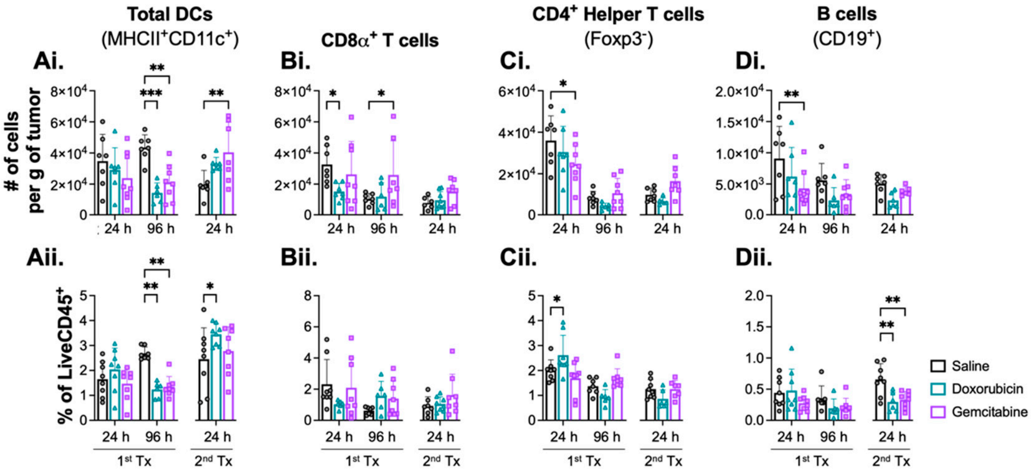

Figure S3. GEM and/or DOX lower the abundance of tumor-infiltrating lymphocytes (TILs) and dendritic cells (DCs) EMT6 tumor microenvironment. 300k parental EMT6 cells were inoculated in the right flanks of BALB/c mice. Mice were injected with GEM (1.2mg), DOX (0.2mg) or saline I.P. on day 14 post inoculation. Tumors were excised 24 and 96 h post initial injection and 24 h post second injection 1 week later. (**A**). Changes in total DC number (**Ai**) and proportion (**Aii**). (**B**). Changes in CD8α+ T cell number (**Bi**) and proportion (**Bii**). (**C**). Changes in CD4+ helper T cell number (**Ci**) and proportion (**Cii**). (**D**). Changes in B cells number (**Di**) and proportion (**Dii**). (n=8) (two-way ANOVA followed by Dunnett's post hoc test for multiple comparison to the control group: * $p < 0.05$, ** $p < 0.01$, *** $p < 0.001$; ROUT Outliers analysis with Q = 1.0%). All points represent mean ± SD.

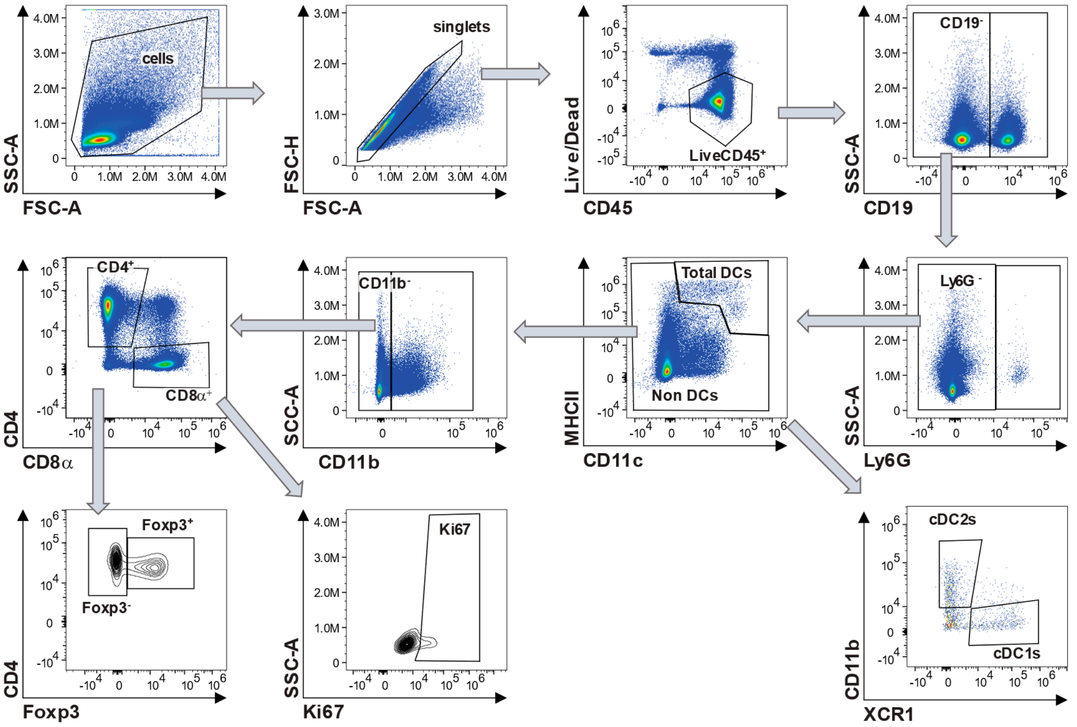

Figure S4. Gating strategy for tdLN flow cytometry analysis in Figure S5. Gating strategy presented for the following immune cell subsets: cDC1s (LiveCD45$^+$CD19$^-$Ly6G$^-$F4/80$^-$CD11c$^+$MHCII$^+$SIRPα$^-$XCR1$^+$) and cDC2s (LiveCD45$^+$CD19$^-$Ly6G$^-$F4/80$^-$CD11c$^+$MHCII$^+$SIRPα$^+$XCR1$^-$).

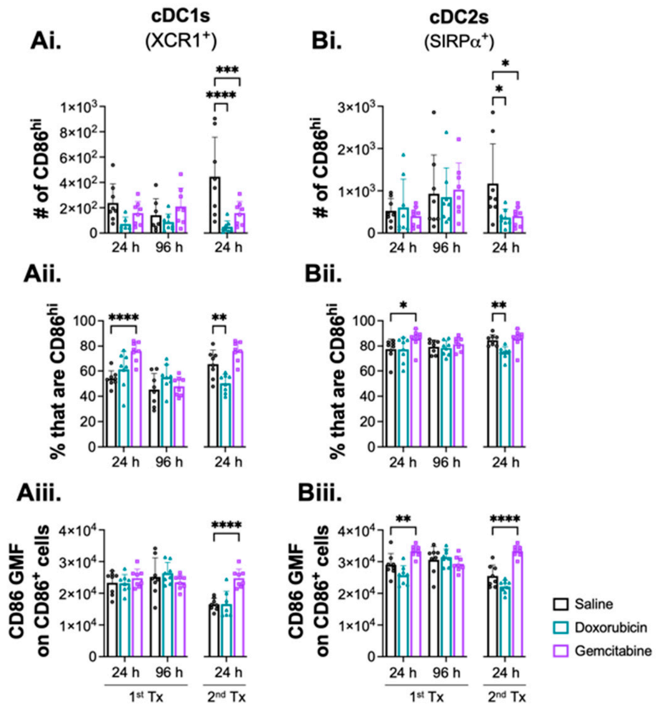

Figure S5: DOX does not induce cDC activation in tdLN. 300k parental EMT6 cells were inoculated in the right flanks of BALB/c mice. Mice were injected with GEM (1.2mg), DOX (0.2mg) or saline I.P. on day 14 post inoculation. tdLN were excised 24 and 96 h post initial injection and 24 h post second injection 1 week later. (**A**). Changes in CD86^{hi} cDC1s (**Ai**), the proportion of cDC1s that are CD86^{hi} (**Aii**), and CD86 GMF on all CD86^{+} cDC1s (**Aiii**). (**B**) Changes in CD86^{hi} cDC2s (**Bi**), the proportion of cDC2s that are CD86^{hi} (**Bii**), and CD86 GMF on all CD86^{+} cDC2s (**Biii**). (n=8) (two-way ANOVA followed by Dunnett's post hoc test for multiple comparison to the control group: * $p < 0.05$, ** $p < 0.01$, *** $p < 0.001$, **** $p < 0.0001$; ROUT Outliers analysis with Q = 1.0%). All points represent mean ± SD.

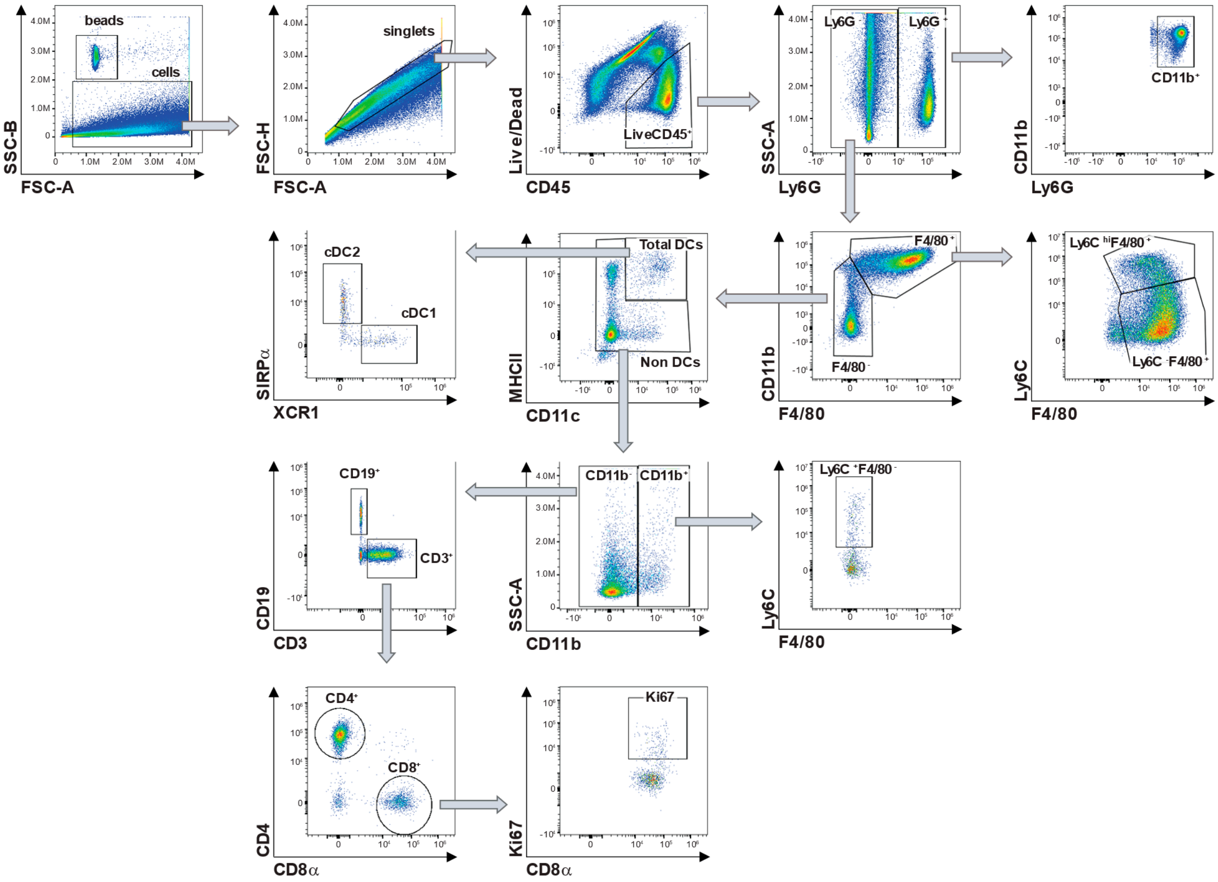

Figure S6. Gating strategy for tumor flow cytometry analysis in Figure 1. Gating strategy presented for the following immune cell subsets: neutrophils (LiveCD45^{+}Ly6G^{+}CD11b^{+}); macrophages (LiveCD45^{+}Ly6G^{-}CD11b^{+}F4/80^{+}Ly6C^{-}); inflammatory monocytes (LiveCD45^{+}Ly6G^{-}CD11b^{+}F4/80^{+}Ly6C^{hi}); monocytes (LiveCD45^{+}Ly6G^{-}F4/80^{-}NonDCs CD11b^{+}Ly6C^{+}); B cells (LiveCD45^{+}Ly6G^{-}F4/80^{-}NonDCs CD11b^{-}CD19^{+}CD3^{-}); T cells (LiveCD45^{+}Ly6G^{-}F4/80^{-}NonDCs CD11b^{-}CD19^{-}CD3^{+}); and dendritic cells (LiveCD45^{+}Ly6G^{-}F4/80^{-}CD11c^{+}MHCII^{+}).

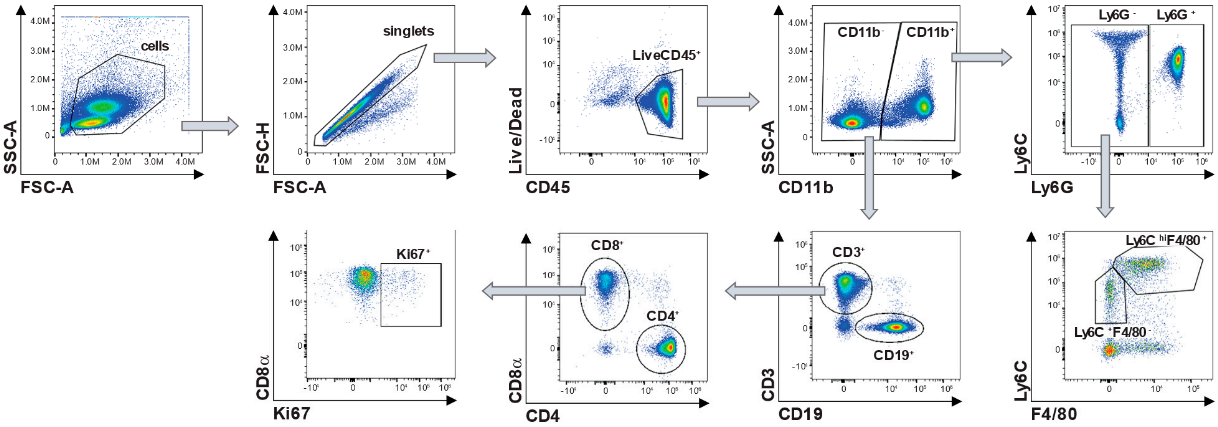

Figure S7. Gating strategy for blood flow cytometry analysis in Figure S8. Gating strategy presented for the following immune cell subsets: neutrophils (LiveCD45$^+$CD11b$^+$Ly6G$^+$); monocytes (LiveCD45$^+$CD11b$^+$Ly6G$^-$Ly6C$^+$F4/80$^-$); inflammatory monocytes (LiveCD45$^+$CD11b$^+$Ly6G$^-$Ly6C^{hi}F4/80$^+$); B cells (LiveCD45$^+$Ly6G$^-$F4/80$^-$NonDCs CD11b$^-$CD19$^+$CD3$^-$); CD8α T cells (LiveCD45$^+$CD11b$^-$CD3$^+$CD19$^-$CD4$^-$CD8α$^+$); and CD4 T cells (LiveCD45$^+$CD11b$^-$CD3$^+$CD19$^-$CD4$^+$CD8α$^-$).

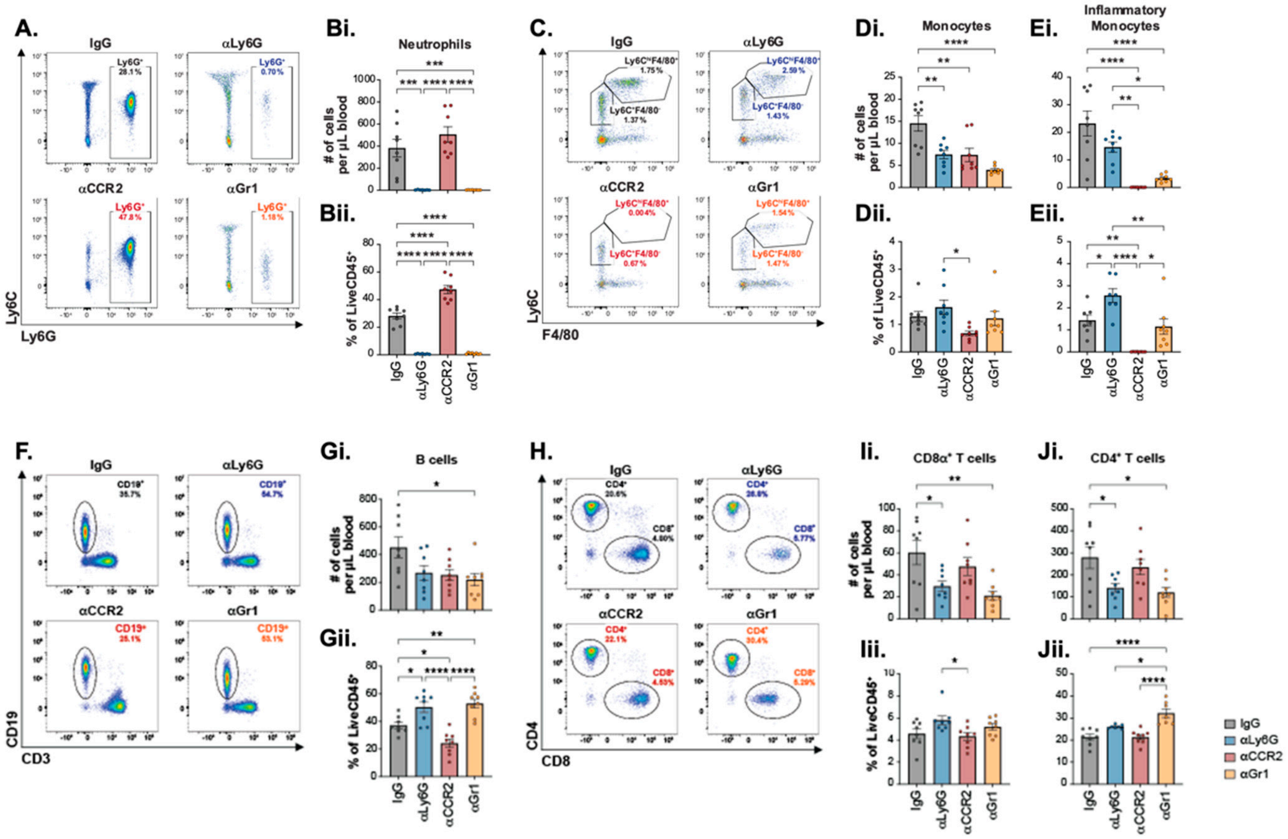

Figure S8. Systemic depletion by myeloid-targeting antibodies. 300k parental EMT6 cells were inoculated in the right flanks of BALB/c mice. Mice were injected with 25µg of Ly6G, Gr1, CCR2, or saline i.p. on day 12 and 14 post inoculation. Blood was taken on d15. **(A)** Representative flow plots showing neutrophil changes in circulation. (**B**). Changes in neutrophil number (**Bi**) and proportion (**Bii**). (**C**). Representative flow plots showing Ly6C^{+} and Ly6C^{hi} monocyte changes in circulation. (**D**) Changes in Ly6C^{+} monocyte number (**Di**) and proportion (**Dii**). (**E**). Changes in Ly6C^{hi} inflammatory monocyte number (**Ei**) and proportion (**Eii**). **(F)** Representative flow plots showing B cell changes in circulation. **(G)**. Changes in B cell number (**Gi**) and proportion (**Gii**). (**H**). Representative flow plots showing CD8α^{+} and CD4^{+} T cell changes in circulation. (**H**) Changes in CD8α^{+} T cell number (**Hi**) and proportion (**Hii**). (**I**). Changes in CD4^{+} T cell number (**Ii**) and proportion (**Iii**). (n=8) (one-way ANOVA followed by Tukey's post hoc test for multiple comparisons: * $p < 0.05$, ** $p < 0.01$, *** $p < 0.001$, **** $p < 0.0001$; ROUT Outliers analysis with Q= 1.0%). All points represent mean ± SD.

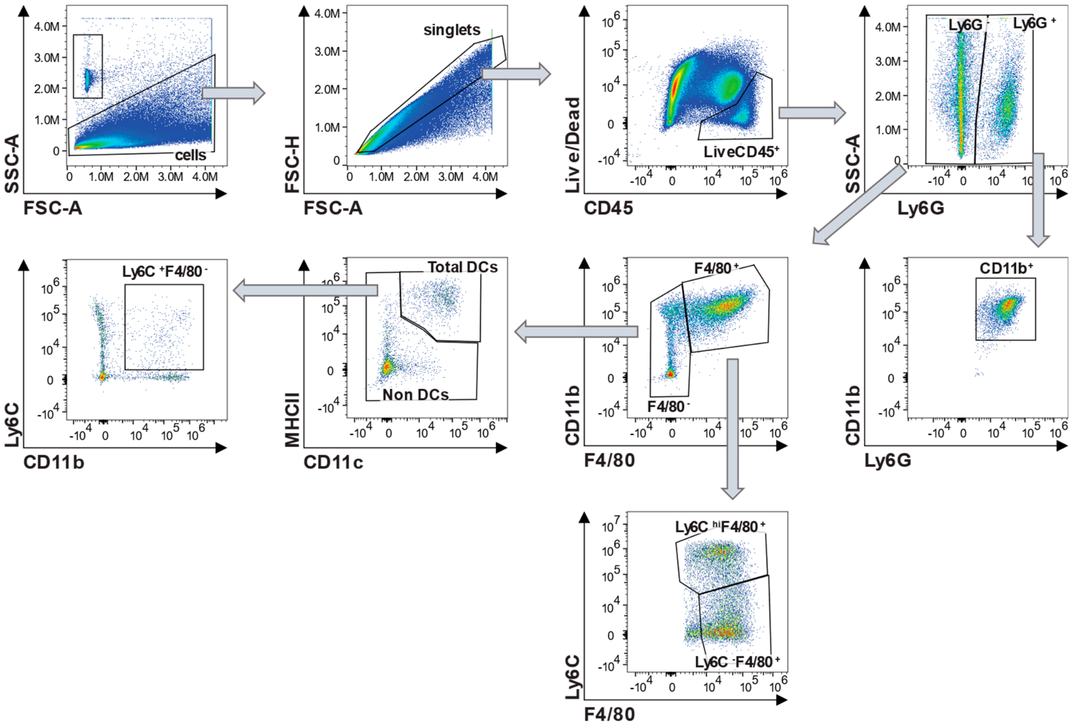

Figure S9. Gating strategy for tumor flow cytometry analysis in Figure S10 and Figure S11. Gating strategy presented for the following immune cell subsets: neutrophils (LiveCD45^{+}Ly6G^{+}CD11b^{+}); inflammatory monocytes (LiveCD45^{+}Ly6G^{-} CD11b^{+}F4/80^{+}Ly6C^{hi}); macrophages (LiveCD45^{+}Ly6G^{-} CD11b^{+}F4/80^{+}Ly6C^{-}).

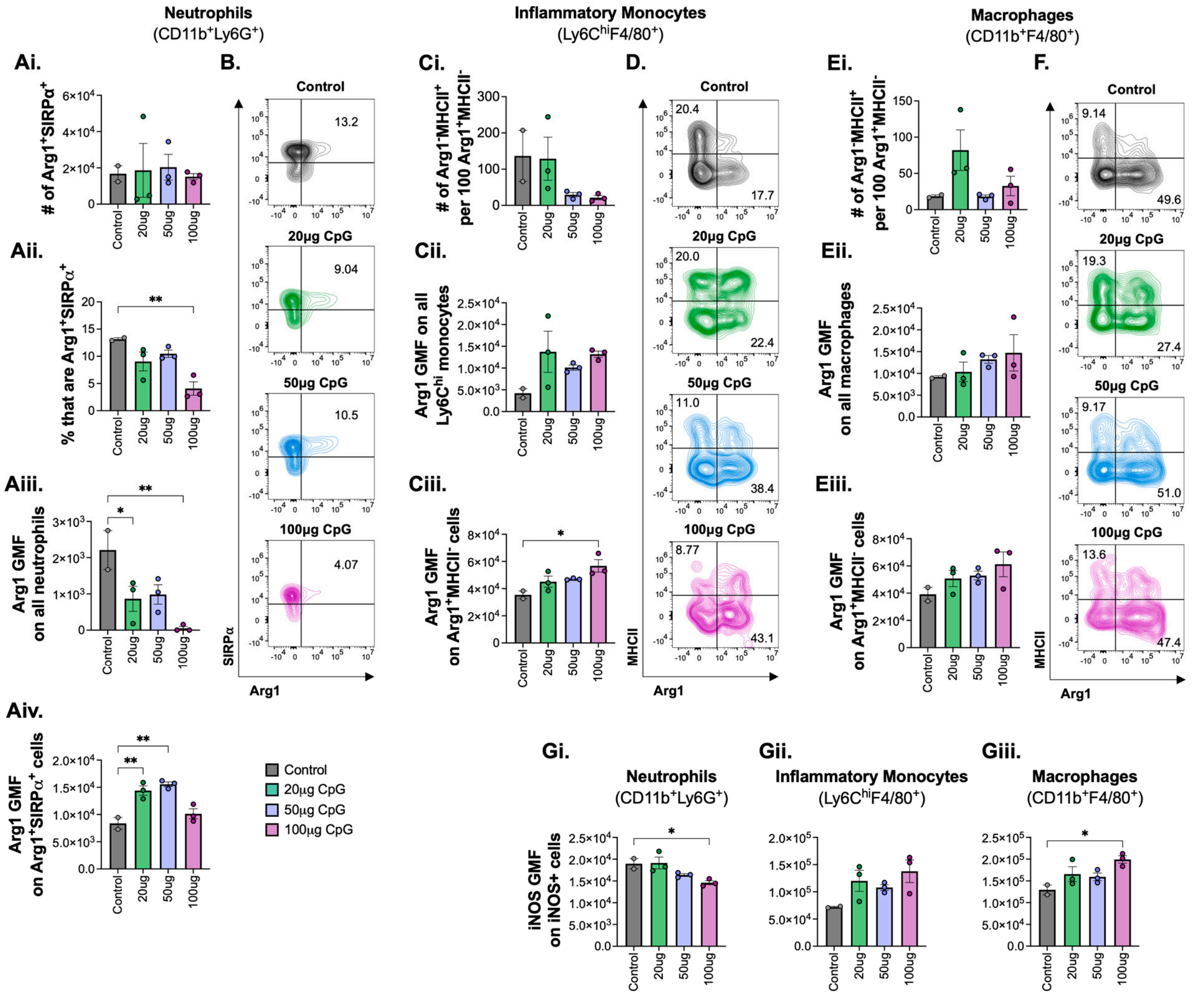

Figure S10. Limited functional myeloid reprogramming by CpG-B. Mice were injected with three doses (20µg, 50µg, and 100µg) of CpG or LAL water i.p. on day 14 post inoculation. Tumors were excised 24 h post injection. (**A**). Changes in the number of Arg1+Sirpa+ neutrophils (**Ai**), proportion of neutrophils that express Arg1+Sirpa+ (**Aii**), Arg1 GMF on total neutrophils (**Aiii**), and Arg1 GMF on Arg1+SIRPα+ neutrophils. (**B**). Representative flow plots for Arg1+Sirpα+ neutrophils. (**C**). Changes in Arg1-MHCII+ per 100 Arg1+MHCII- inflammatory monocytes (**Ci**), Arg1 GMF on total inflammatory monocytes (**Cii**), and Arg1 GMF on Arg1+MHCII- inflammatory monocytes (**Ciii**). (**D**). Representative flow plots for Arg1-MHCII+ and Arg1+MHCII- inflammatory monocytes. (**E**). Changes in Arg1+MHCII+ per 100 Arg1+MHCII- macrophages (**Ei**), Arg1 GMF on total macrophages (**Eii**), and Arg1 GMF on Arg1+MHCII- macrophages (**Eiii**). (**F**). Representative flow plots for Arg1-MHCII+ and Arg1+MHCII- macrophages. (**G**) iNOS GMF on iNOS+ neutrophils (**Gi**), iNOS+ inflammatory monocytes (**Gii**), and iNOS+ macrophages (**Giii**). (n=2-3) (one-way ANOVA followed by Dunnett's post hoc test for multiple comparison to the control group: * $p < 0.05$, ** $p < 0.01$; ROUT Outliers analysis with Q = 1.0%). All points represent mean ± SD. Representative flow plots were picked from down sampled data concatenated on the LiveCD45+ gate.

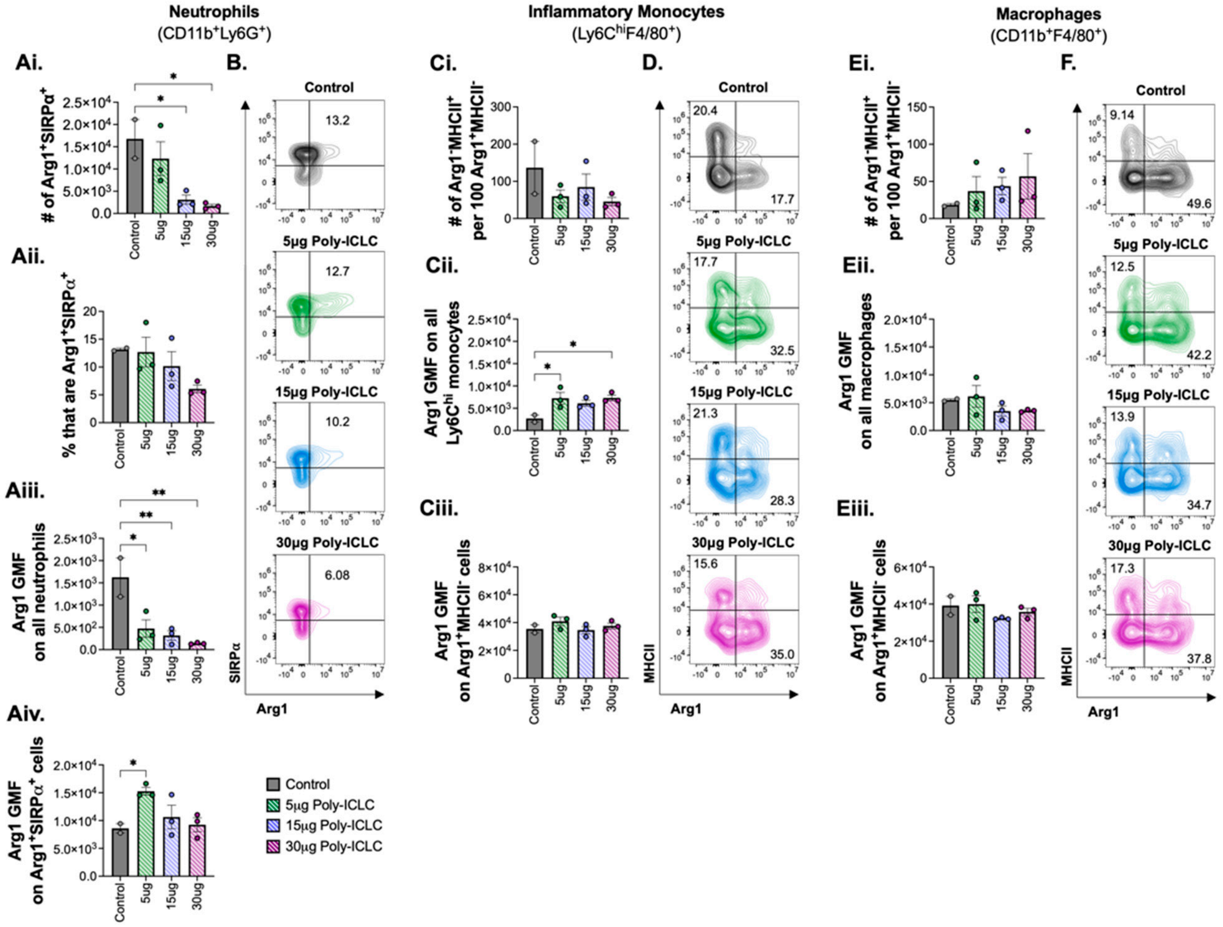

Figure S11. Poly-ICLC impact on myeloid functional activity in the TME. Mice were injected with three doses (5µg, 15µg, 30µg) of Poly-ICLC or LAL water i.p. on day 14 post inoculation. Tumors were excised 24 h post injection. (**A**). Changes in the number of $Arg1^{+}Sirpa^{+}$ neutrophils (**Ai**), proportion of neutrophils that express $Arg1^{+}Sirpa^{+}$ (**Aii**), Arg1 GMF on total neutrophils (**Aiii**), and Arg1 GMF on $Arg1^{+}SIRP\alpha^{+}$ neutrophils. **(B)** Representative flow plots for $Arg1^{+}Sirp\alpha^{+}$ neutrophils. **(C)** Changes in $Arg1^{-}MHCII^{+}$ per 100 $Arg1^{+}MHCII^{-}$ inflammatory monocytes (**Ci**), Arg1 GMF on total inflammatory monocytes (**Cii**), and Arg1 GMF on $Arg1^{+}MHCII^{-}$ inflammatory monocytes (**Ciii**). **(D)** Representative flow plots for $Arg1^{-}MHCII^{+}$ and $Arg1^{+}MHCII^{-}$ inflammatory monocytes. (**E**) Changes in $Arg1^{+}MHCII^{+}$ per 100 $Arg1^{+}MHCII^{-}$ macrophages (**Ei**), Arg1 GMF on total macrophages (**Eii**), and Arg1 GMF on $Arg1^{+}MHCII^{-}$ macrophages (**Eiii**). **(F)** Representative flow plots for $Arg1^{-}MHCII^{+}$ and $Arg1^{+}MHCII^{-}$ macrophages. (n=2-3) (one-way ANOVA followed by Dunnett's post hoc test for multiple comparison to the control group: * $p < 0.05$, ** $p < 0.01$; ROUT Outliers analysis with Q = 1.0%). All points represent mean ± SD. Representative flow plots were picked from down sampled data concatenated on the $LiveCD45^{+}$ gate.

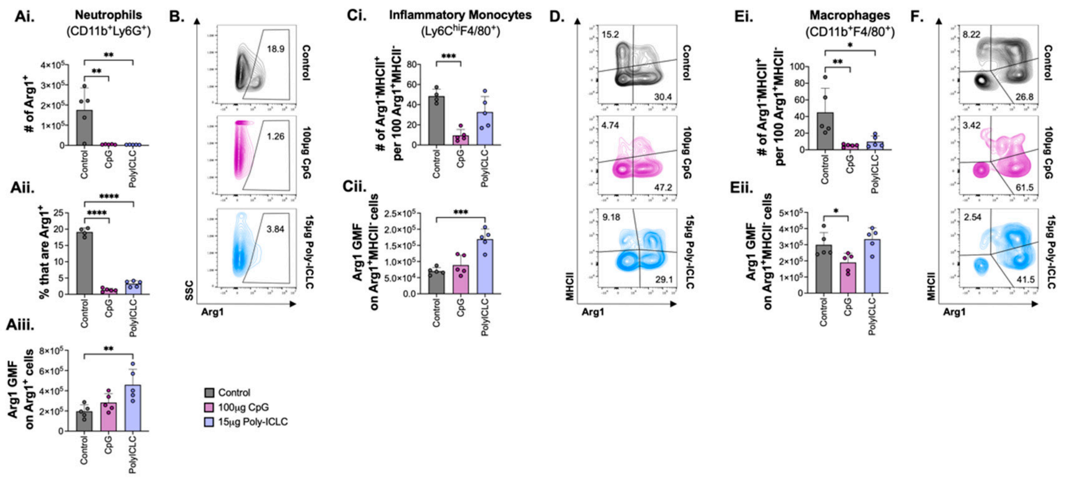

Figure S12. TLR agonism fails to promote durable pro-inflammatory myeloid phenotypes in the 4T1 TME. Mice were injected with 100µg CpG, 15µg Poly-ICLC, or LAL water i.p. on day 14 post inoculation. Tumors were excised 24 h post injection. (**A**). Changes in the number of Arg1$^+$ neutrophils (**Ai**), proportion of neutrophils that express Arg1$^+$ (**Aii**), Arg1 GMF on Arg1$^+$ neutrophils (**Aiii**). **(B)** Representative flow plots for Arg1$^+$ neutrophils. **(C)** Changes in the ratio of Arg1$^-$MHCII$^+$ per 100 Arg1$^+$MHCII$^-$ inflammatory monocytes (**Ci**) and Arg1 GMF on Arg1$^+$MHCII$^-$ inflammatory monocytes (**Cii**). **(D)** Representative flow plots for Arg1$^-$MHCII$^+$ and Arg1$^+$MHCII$^-$ inflammatory monocytes. (**E**) Changes in the ratio of Arg1$^+$MHCII$^+$ per 100 Arg1$^+$MHCII$^-$ macrophages (**Ei**) and Arg1 GMF on Arg1$^+$MHCII$^-$ macrophages (**Eii**). **(F)** Representative flow plots for Arg1$^-$MHCII$^+$ and Arg1$^+$MHCII$^-$ macrophages. (n=5) (one-way ANOVA followed by Dunnett's post hoc test for multiple comparison to the control group: * $p < 0.05$, ** $p < 0.01$; ROUT Outliers analysis with Q = 1.0%). All points represent mean ± SD.

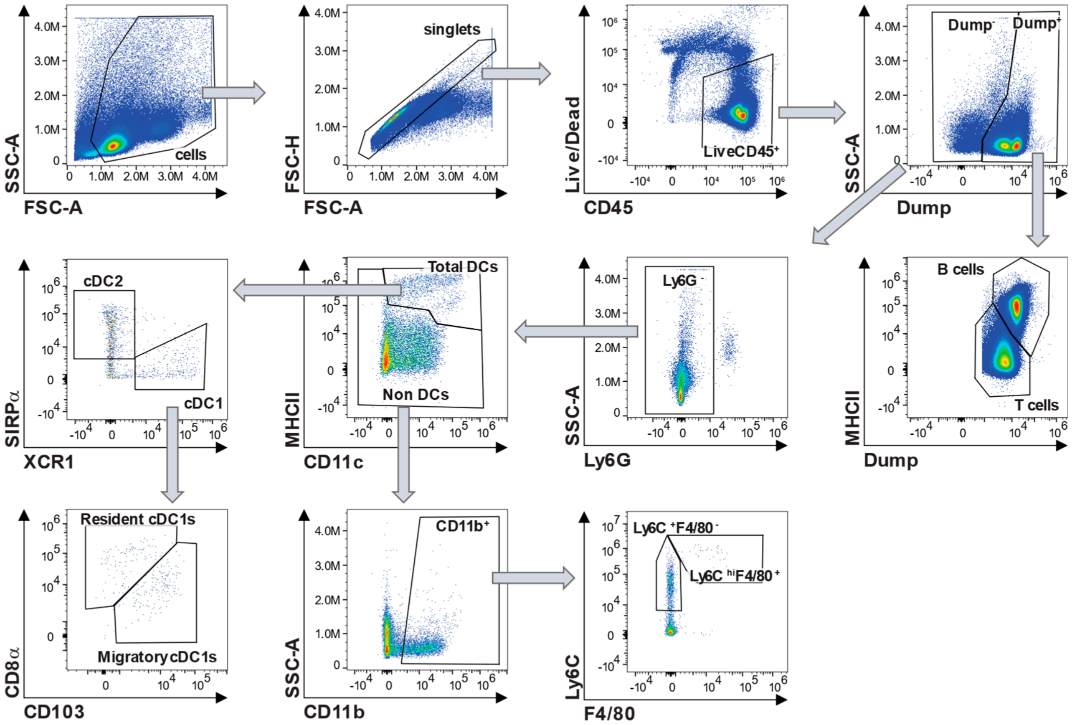

Figure S13. Gating strategy for tdLN flow cytometry analysis in Figure S14, Figure S15, and S16. Gating strategy presented for the following immune cell subsets: B cells (LiveCD45^{+}Dump MHCII^{+}); T cells (LiveCD45^{+}Dump MHCII^{-}); dendritic cells (LiveCD45^{+}Dump-Ly6G^{-}CD11c^{+}MHCII^{+}); monocytes (LiveCD45^{+}Dump-Ly6G^{-}Non DCs CD11b^{+}Ly6C^{+}F4/80^{-}); and inflammatory monocytes (LiveCD45^{+}Dump-Ly6G^{-}Non DCs CD11b^{+}Ly6C^{hi}F4/80^{+}). The dump gate included CD19 and CD3 markers.

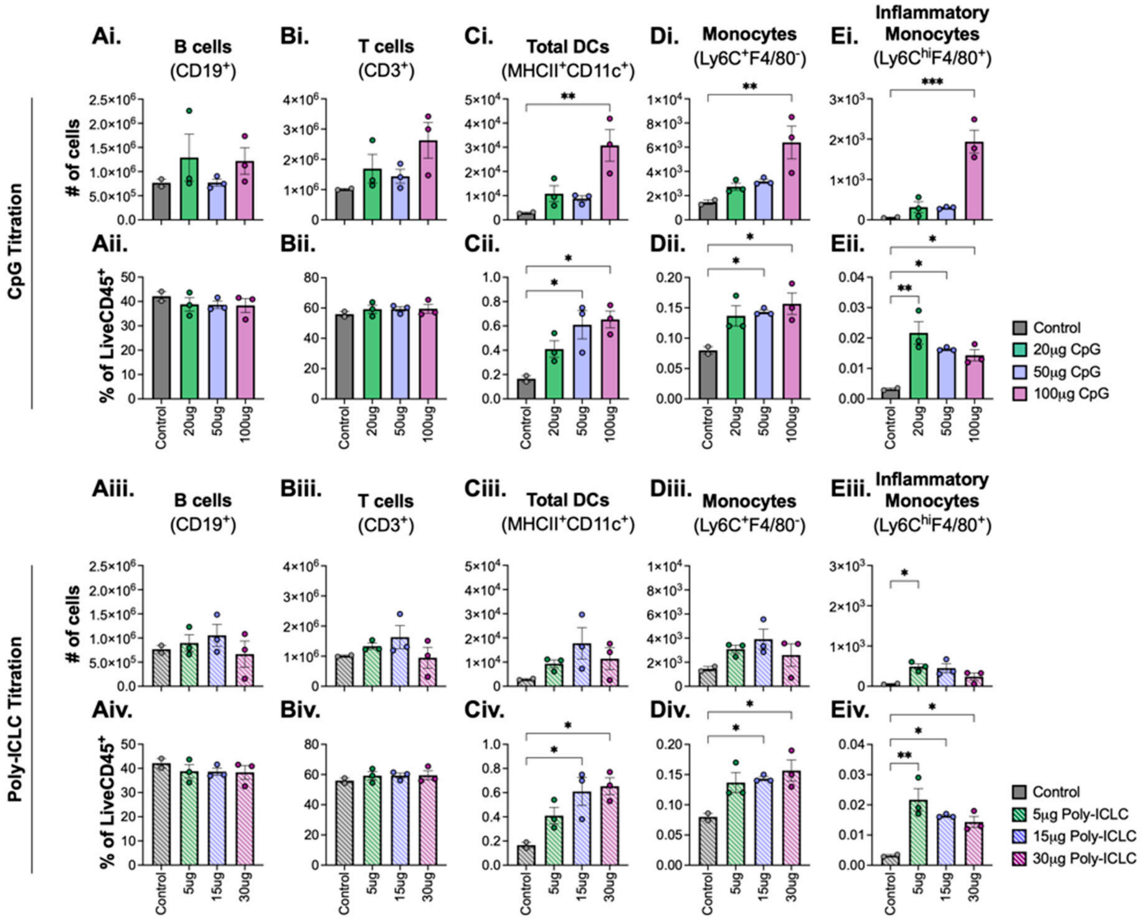

Figure S14. TLR agonist- and dose-dependent modulation of immune populations in the tdLN. Mice were injected with three doses (20µg, 50µg, and 100µg) of CpG, three doses (5µg, 15µg, and 30µg) of Poly-ICLC, or LAL water i.p. on day 14 post inoculation. TdLNs were excised 24 h post injection. (**A**). Changes in B cell numbers (**Ai, Aiii**) and proportion (**Aii, Aiv**). (**B**). Changes in T cell numbers (**Bi, Biii**) and proportion (**Bii, Biv**). (**C**). Changes in total DC numbers (**Ci, Ciii**) and proportion (**Cii, Civ**). (**D**). Changes in monocyte numbers (**Di, Diii**) and proportion (**Dii, Div**). **(E)** Changes in inflammatory monocyte numbers (**Ei, Eiii**) and the proportion (**Eii, Eiv**). **(**n=2-3) (one-way ANOVA followed by Dunnett's post hoc test for multiple comparison to the control group: * $p < 0.05$, ** $p < 0.01$, *** $p < 0.001$; ROUT Outliers analysis with Q = 1.0%). All points represent mean ± SD.

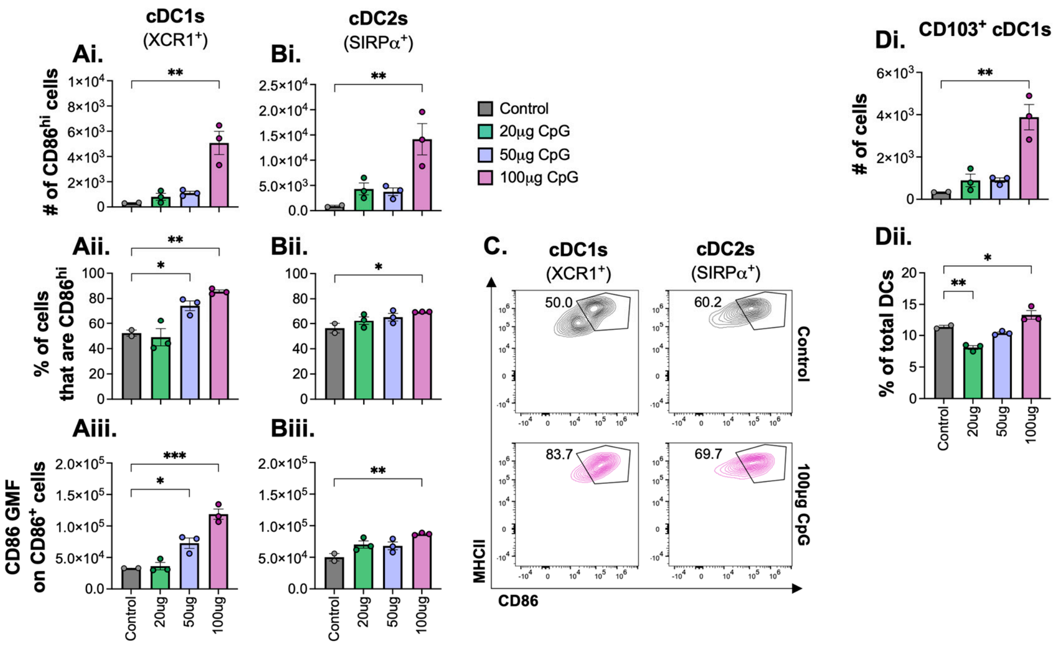

Figure S15. High dose CpG-B drives cDC activation and mobilization. Mice were injected with three doses (20µg, 50µg, and 100µg) of CpG or LAL water i.p. on day 14 post inoculation. TdLNs were excised 24 h post injection. (**A**). Changes in activated cDC1 numbers (**Ai**), proportion of cDC1s that are activated (**Aii**), and CD86 GMF (**Aiii**). (**B**). Changes in activated cDC2 numbers (**Bi**), proportion of cDC2s that are activated (**Bii**), and CD86 GMF (**Biii**). (**C**). Representative flow plots for cDC activation for control (black) and 100µg CpG (pink). (**D**) Changes in migratory cDC1 numbers (**Di**) and the proportion of total DCs that are CD103+ cDC1s (**Dii**). (n=2-3) (one-way ANOVA followed by Dunnett's post hoc test for multiple comparison to the control group: * $p < 0.05$, ** $p < 0.01$, *** $p < 0.001$; ROUT Outliers analysis with Q = 1.0%). All points represent mean ± SD.

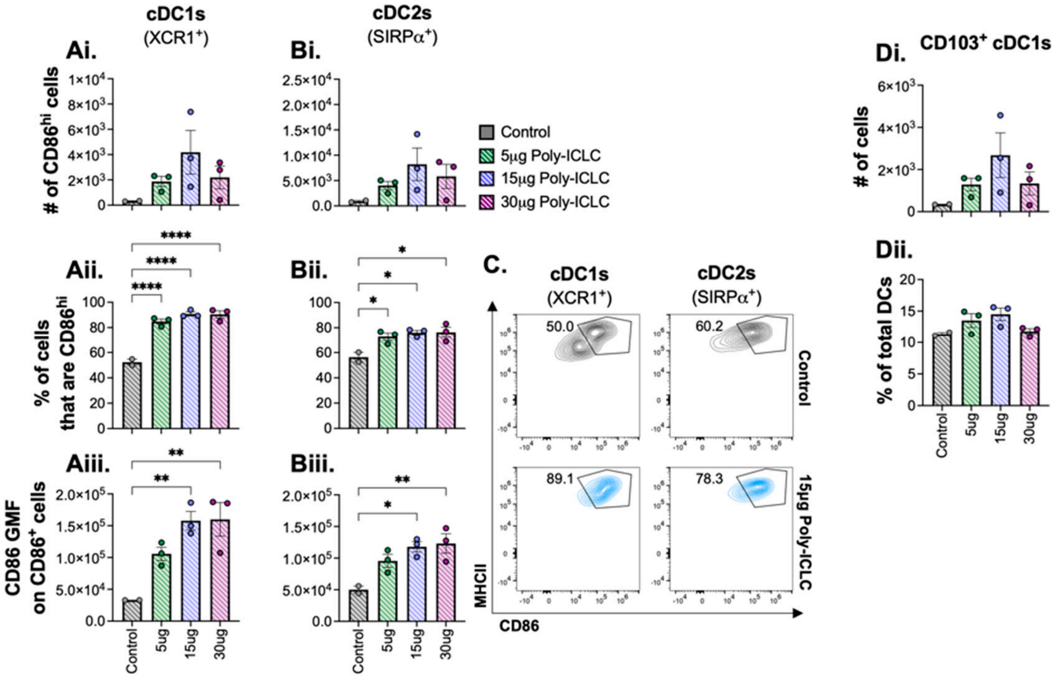

Figure S16. Poly-ICLC driven cDC activation and migration. Mice were injected with three doses (5μg, 15μg, and 30μg) of Poly-ICLC or LAL water i.p. on day 14 post inoculation. TdLNs were excised 24 h post injection. (**A**). Changes in activated cDC1 numbers (**Ai**), proportion of cDC1s that are activated (**Aii**), and CD86 GMF (**Aiii**). (**B**). Changes in activated cDC2 numbers (**Bi**)**,** proportion of cDC2s that are activated (**Bii**), and CD86 GMF (**Biii**). **(C)** Representative flow plots for cDC activation for control (black) and 15μg Poly-ICLC (blue). (**D**) Changes in migratory cDC1 numbers (**Di**) and the proportion of total DCs that are CD103^{+} cDC1s (**Dii**). **(**n=2-3) (one-way ANOVA followed by Dunnett's post hoc test for multiple comparison to the control group: * $p < 0.05$, ** $p < 0.01$ **** $p < 0.0001$; ROUT Outliers analysis with Q = 1.0%). All points represent mean ± SD.

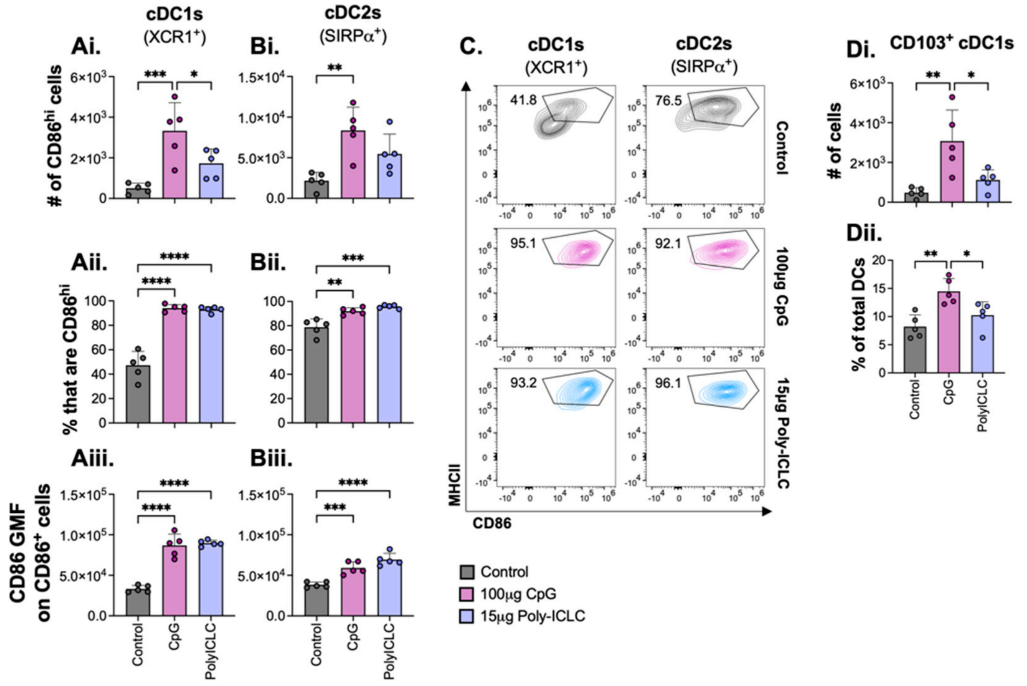

Figure S17. TLR driven cDC activation and mobilization recapitulated in 4T1 tumor-bearing mice. Mice were injected with 100µg of CpG, 15µg of Poly-ICLC or LAL water i.p. on day 14 post inoculation. TdLNs were excised 24 h post injection. (**A**). Changes in activated cDC1 numbers (**Ai**), proportion of cDC1s that are activated (**Aii**), and CD86 GMF (**Aiii**). (**B**). Changes in activated cDC2 numbers (**Bi**), proportion of cDC2s that are activated (**Aii**), and CD86 GMF (**Biii**). (**C**) Representative flow plots for cDC activation for control (black), CpG (pink) and Poly-ICLC (blue). (**D**) Changes in migratory cDC1 numbers (**Di**) and the proportion of total DCs that are $CD103^+$ cDC1s (**Dii**). (**E**) Representative flow plots for $CD103^+$ cDC1s for control, 100µg CpG, and 15µg Poly-ICLC. (n=5) (one-way ANOVA followed by Tukey's post hoc test for multiple comparisons: * $p < 0.05$, ** $p < 0.01$, *** $p < 0.001$, **** $p < 0.0001$; ROUT Outliers analysis with Q = 1.0%). All points represent mean ± SD.

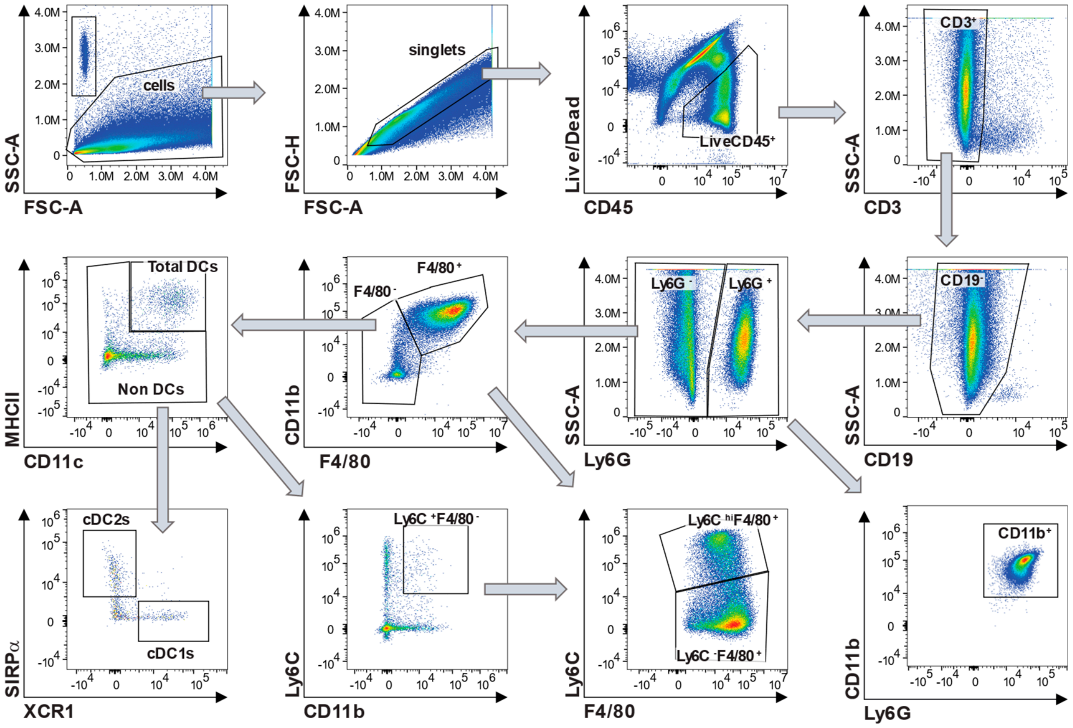

Figure S18. Gating strategy for tumor flow cytometry analysis in Figure 2. Gating strategy presented for the following immune cell subsets: inflammatory monocytes (LiveCD45^{+}CD3^{-}CD19^{-}Ly6G^{-}CD11b^{+}F4/80^{+}Ly6C^{hi}); macrophages (LiveCD45^{+}CD3^{-}CD19^{-}Ly6G^{-}CD11b^{+}F4/80^{+}Ly6C^{-}); and neutrophils (LiveCD45^{+}CD3^{-}CD19^{-}Ly6G^{+}CD11b^{+}).

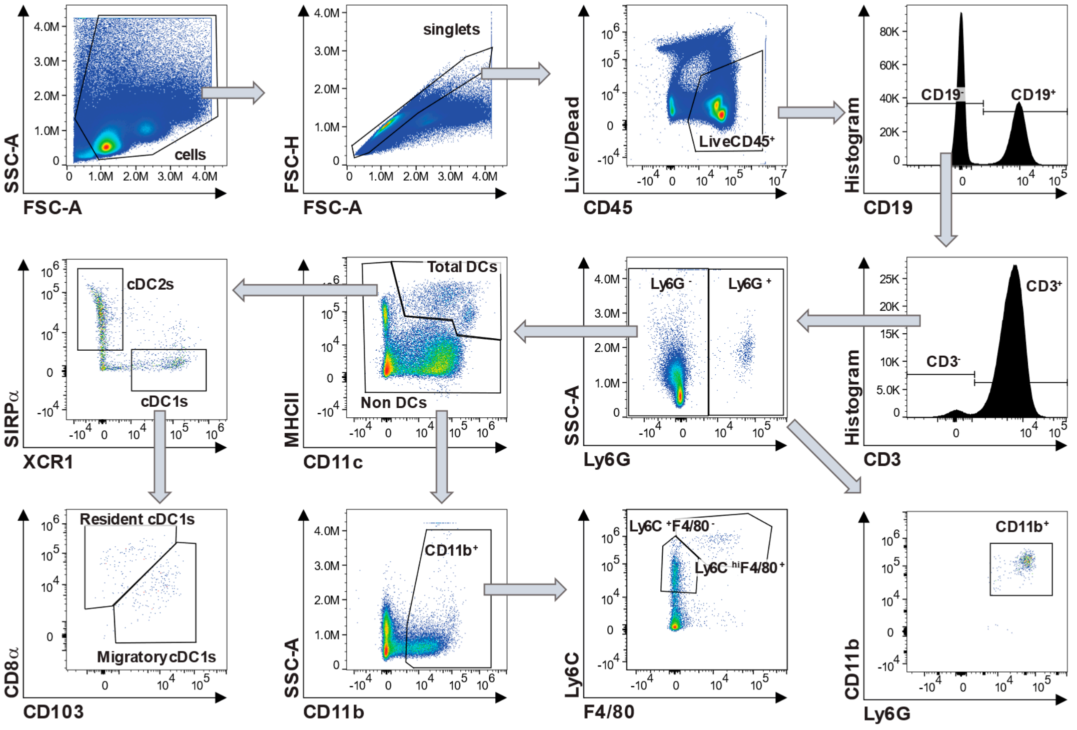

Figure S19. Gating strategy for tdLN flow cytometry analysis in Figure S20. Gating strategy presented for the following immune cell subsets: cDC1s (LiveCD45$^+$CD19$^-$CD3$^-$Ly6G$^-$CD11c$^+$MHCII$^+$ SIRPα$^-$XCR1$^+$); cDC2s (LiveCD45$^+$CD19$^-$CD3$^-$Ly6G$^-$CD11c$^+$MHCII$^+$ SIRPα$^+$XCR1$^-$). Representative ZsG gating was shown in Figure S20.

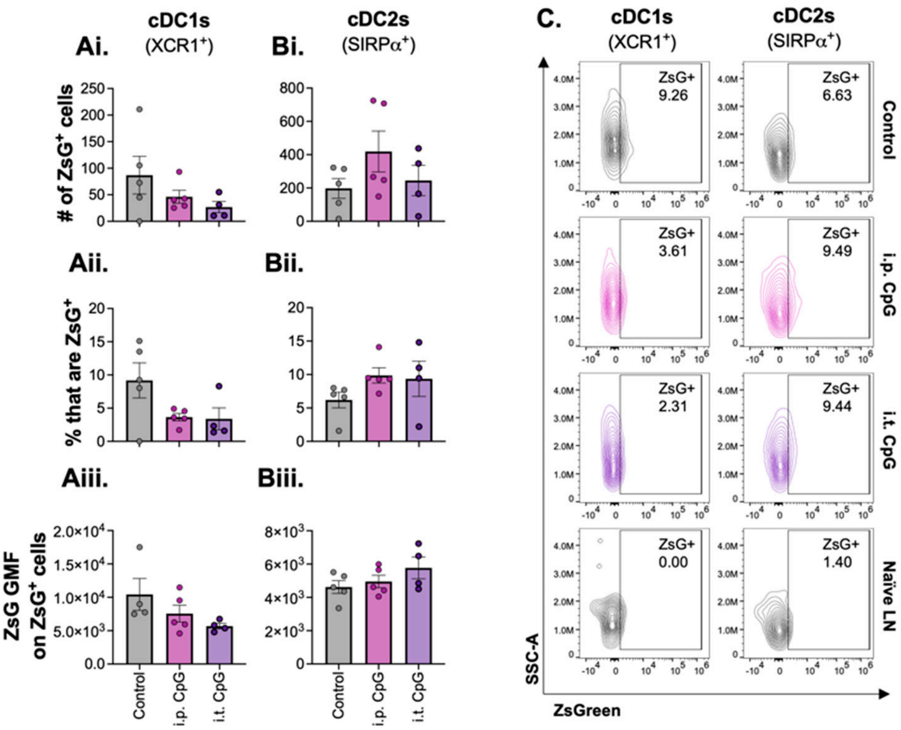

Figure S20. Neither i.p. nor i.t. CpG administration increase tumor-antigen drainage to the tdLN. 300k ZsG+ EMT6 cells were inoculated in the right flanks of BALB/c mice. Mice were injected with CpG (100µg) i.p. or i.t. on day 14 post inoculation. TdlNs were excised 24 h post injection. (**A**). Changes in the number of ZsG+ cDC1s (**Ai**), the proportion of cDC1s that are ZsG+ (**Aii**), and the ZsG GMF on ZsG+ cDC1s (**Aiii**). (**B**). Changes in the number of ZsG+ cDC2s (**Bi**), the proportion of cDC2s that are ZsG+ (**Bii**), and the ZsG GMF on ZsG+ cDC2s (**Biii**). (C) Representative flow plots. Lymph nodes (LN) taken from a naïve mouse were used for ZsG FMOs. (n=4-5) (one-way ANOVA followed by Tukey's post hoc test for multiple comparisons: no significance; ROUT Outliers analysis with Q = 1.0%). All points represent mean ± SD.

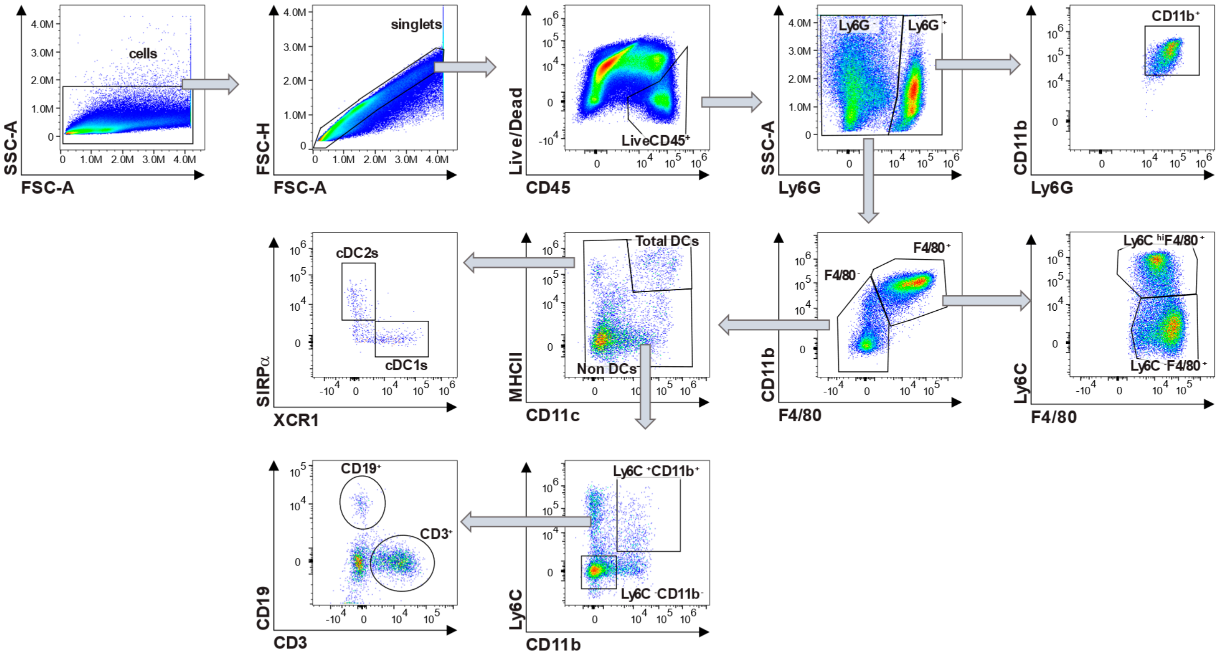

Figure S21. Gating strategy for tumor flow cytometry analysis in Figure 4 & 5. Gating strategy presented for the following immune cell subsets: LiveCD45$^+$; neutrophils (LiveCD45$^+$Ly6G$^+$CD11b$^+$); macrophages (LiveCD45$^+$Ly6G$^-$CD11b$^+$F4/80$^+$Ly6C$^-$); inflammatory monocytes (LiveCD45$^+$Ly6G$^-$CD11b$^+$F4/80$^+$Ly6C^{hi}); monocytes (LiveCD45$^+$Ly6G$^-$F4/80$^-$NonDCs CD11b$^+$Ly6C$^+$); B cells (LiveCD45$^+$Ly6G$^-$F4/80$^-$NonDCs Ly6C$^-$CD11b$^-$CD19$^+$CD3$^-$); T cells (LiveCD45$^+$Ly6G$^-$F4/80$^-$NonDCs Ly6C$^-$CD11b$^-$CD19$^-$CD3$^+$); and dendritic cells (LiveCD45$^+$Ly6G$^-$F4/80$^-$CD11c$^+$MHCII$^+$).

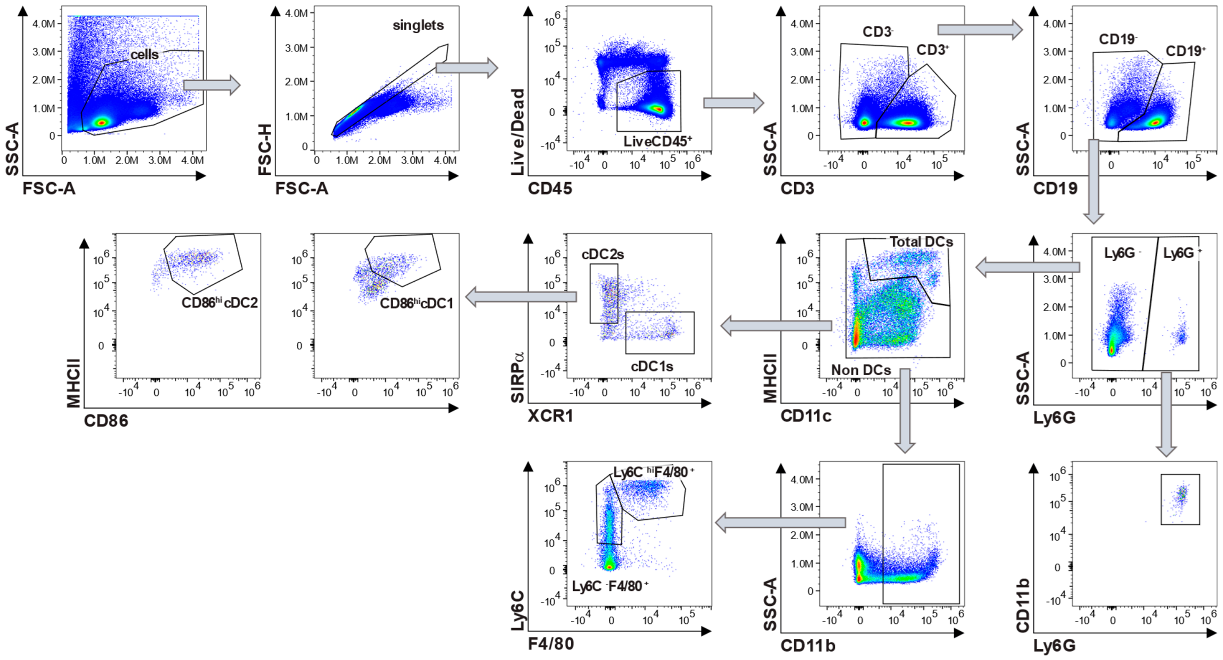

Figure S22. Gating strategy for tdLN flow cytometry analysis in Figure 6. Gating strategy presented for the following immune cell subsets: cDC1s (LiveCD45^{+}CD3^{-}CD19^{-}Ly6G^{-}F4/80^{-}CD11c^{+}MHCII^{+}SIRPα^{-}XCR1^{+}); cDC2s (LiveCD45^{+}CD3^{-}CD19^{-}Ly6G^{-}F4/80^{-}CD11c^{+}MHCII^{+}SIRPα^{+}XCR1^{-}). CD86^{hi} gate also shown for both cDC1 and cDC2.

Feature/Treatment	EMT6	4T1
GEM Effect	Systemic myeloablation; fails to reduce intratumoral isMCs.	Systemic myeloablation; fails to reduce intratumoral isMCs [42].
DOX Effect	Transient neutrophil reduction; no cDC activation.	No data.
Ab-Mediated Depletion	**Tolerated**; effective systemic/intratumoral depletion (though transient).	**Lethal toxicity**; regimens proved fatal even after dose titration.
TLR Agonism (CpG)	Failed to reprogram isMCs; induced broad immune cell loss (ablative).	Failed to reprogram isMCs; induced broad immune cell loss (ablative), Arg1+ status maintained.
TLR Agonism (Poly-ICLC)	Dose-dependent reduction in Arg1+ neutrophils, but no stable phenotypic shift.	No meaningful reprogramming observed.
cDC Mobilization	Robust cDC activation/migration to tdLN with high-dose CpG.	Recapitulated high-dose CpG response (100 µg).
T-FUS Interaction	T-FUS is the dominant driver; no additive benefit from Ab/TLR.	Synergy with GEM reported previously [41].

Table S1: Summary of observed effects on myeloid cells in the two models of TNBC
